# Age-period-cohort analysis of cardiovascular disease trends in middle-aged and older adults: cross-country comparison across HRS, ELSA, SHARE, and CHARLS

**DOI:** 10.7189/jogh.15.04260

**Published:** 2025-09-12

**Authors:** Jiajia Li, Shiqi Lin, Heming Pei, Guilan Xie, Lijun Pei, Gong Chen

**Affiliations:** 1 Institute of Population Research, Peking University, Beijing, China; 2 School of Humanities and Social Science, Fuzhou University, Fujian, China; 3 Butler Columbia Aging Center, Columbia University Mailman School of Public Health, New York, New York, USA; 4 Department of Epidemiology, Columbia University Mailman School of Public Health, New York, New York, USA

## Abstract

**Background:**

Cardiovascular disease (CVD) is a leading cause of death globally, while the dynamics of CVD risk across different age groups, periods, and birth cohorts remain unclear. This study investigates how age, period, and cohort effects contribute to CVD risk across regions differently.

**Methods:**

We employed a repeated cross-sectional design, analysing data from four large longitudinal surveys in the USA, UK, Europe, and China. A hierarchical age-period-cohort analysis was conducted using Bayesian inference through the integrated nested Laplace approximation to model the effects of age, period, and cohort on CVD risk across these regions. Subgroup analyses were also conducted to examine the moderation effects of social-demographic factors.

**Results:**

CVD risk increases with age across all regions, peaking at age 75 in China while continuously rising in other areas. Period effects showed a significant increase in CVD risk over time in the USA, UK, and China, while a decline was observed in Europe after 2017. More recent birth cohorts showed a lower CVD risk, especially in the USA and UK. In China, the decrease in risk among recent cohorts was less pronounced. Gender, marital status, education, rural residence, and smoking moderated CVD risk trends across regions.

**Conclusions:**

This study highlights the importance of age, period, and cohort effects in understanding regional differences in CVD risk among middle-aged and older adults. Findings suggest that public health interventions should be tailored to specific regions and demographic groups to reduce CVD burden effectively.

Cardiovascular disease (CVD), including ischemic heart disease and stroke, is the leading cause of death worldwide. An estimated 20.5 million deaths in 2021 were attributed to CVD, accounting for one-third of global deaths [1]. The World Health Organization (WHO) has prioritised the reduction of non-communicable diseases (NCDs), including CVD, through strategies aimed at improving prevention, treatment, and health equity across regions [2]. As the global population ages, understanding the dynamics of CVD risk is essential for achieving the WHO’s goal of reducing premature mortality from NCDs by 2030 [3]. Ageing populations are at increased risk for CVD due to the accumulation of common risk factors over the life course, such as obesity, hypertension, high cholesterol, smoking, and diabetes [3,4]. The age-period-cohort (APC) framework is essential to understanding how CVD risks evolve when countries adopt different policies at different times. It examines the interplay of age effects (physiological ageing), period effects (calendar-year influences), and cohort effects (shared early-life conditions), providing insights into how CVD risk results primarily from ageing, policy shifts, or differences between cohorts [5].

Previous studies have found that CVD risk increases with age due to age-related physiological changes such as arterial stiffening and the accumulation of atherosclerotic plaques [3]. Additionally, period effects are essential in understanding temporal shifts in CVD risk, as evidenced by the association between strengthened alcohol-related public health policies and the long-term decline in CVD mortality [6]. Improvements in health care systems and changes in lifestyle trends, such as reductions in smoking rates and better hypertension management, have also significantly influenced the decline in CVD mortality rates [7]. Cohort effects also contribute to CVD risk, with early-life exposures, such as famine, being linked to a notably higher risk of CVD in later life [8]. Individuals who experienced more adversities in childhood exhibit higher CVD risk in adulthood compared to those born in more favourable conditions [9]. Hence, CVD risk is not only individual behaviour but also the interplay between age, period, and cohort effects within broader socio-historical contexts [5].

Previous studies have highlighted substantial cross-country variation in CVD-related mortality [10], with these disparities increasingly concentrated in specific geographic regions. In high-income countries such as the USA and the UK, where health care systems are well-established, recent cohorts have demonstrated lower CVD mortality, attributed to improvements in early-life health and more effective management of risk factors [11]. However, despite these advances, the rate of decline in CVD mortality has significantly slowed in recent years, particularly among individuals aged 35–74, likely due to the rising prevalence of obesity and other emerging risk factors [12]. In contrast, China, undergoing rapid urbanisation and economic transformation, is experiencing increasing CVD risks, with growing exposure to risk factors such as obesity, hypertension, and the consumption of unhealthy non-staple foods [13]. Urban-rural disparities in CVD risk are particularly pronounced in developing countries; between 1991 and 2011, rapid urbanisation in China led to heightened CVD risks among middle-aged and older adults, with women in less urbanised areas being disproportionately affected [14].

Despite the substantial body of research on CVD, most earlier APC studies are single-country analyses, and those few multi-country studies relied on aggregate mortality rather than harmonised micro-data [5,15]. Previous studies have identified several key risk factors for CVD, including behavioural, metabolic, and socio-economic factors, grip strength, and environmental exposures like pollution [16]. Biological sex and gender roles shape CVD patterns [17], and lower education reduces disease-free life expectancy [18]. However, it remains unclear whether regional social gradients in CVD arise from early-life cohort exposures, contemporary period conditions, or individual level age distribution of risk factors. Disentangling these temporal components can guide policymakers in decision making.

This study aims to fill this gap by applying a Bayesian hierarchical APC model to harmonised data from four large ageing surveys, covering 1992–2023. It examines the complex interplay of age, period, and cohort effects on CVD risk across high-income and middle-income regions. By identifying high-risk population groups and exploring sociodemographic moderators of CVD risk. The findings can inform region-specific public-health strategies aligned with WHO targets for healthy ageing.

## METHODS

### Data and samples

This study utilised a repeated cross-sectional design, using data from four large longitudinal surveys: the Health and Retirement Study (HRS), the English Longitudinal Study of Ageing (ELSA), the Survey of Health, Ageing, and Retirement in Europe (SHARE), and the China Health and Retirement Longitudinal Study (CHARLS). These surveys provided comprehensive data on ageing populations across diverse geographic regions, encompassing a wealth of information on health, socio-economic status, and demographic characteristics. The survey designs have been well-documented in previous publications [19–22].

To capture temporal trends, we derived data from multiple waves of each survey. Specifically, this study included HRS data from 1992 to 2022 (Waves 1–16), ELSA data from 2002 to 2023 (Waves 1–10), SHARE data from 2004 to 2021 (Waves 1–9), and CHARLS data from 2011 to 2020 (Waves 1–5). To conduct cross-country comparisons, we also utilised the Harmonized HRS Version D, Harmonized ELSA Version G.3, Harmonized SHARE Version F, and Harmonized CHARLS Version D data sets developed by the Gateway to Global Aging Data project [23], which harmonised instrument differences while allowing each region to retain its own sampling frame and context.

We excluded individuals under the age of 50 from our analysis to focus on the middle-aged and older population, which is at a greater risk of CVD. Additionally, we also excluded participants with missing CVD information. The final sample sizes comprised 283 107 observations for HRS, 94 999 for ELSA, 443 723 for SHARE, and 76 689 for CHARLS.

### Outcome measures

The primary outcome of this study was CVD. We identified CVD in participants from the HRS, ELSA, SHARE, and CHARLS surveys based on self-reported physician diagnoses of heart disease, which included angina, heart attack, congestive heart failure, and other heart-related conditions, as well as stroke. Specifically, participants were asked the following questions: ‘Have you ever been told by a doctor that you have had a heart attack, angina, coronary heart disease, heart failure, or other heart problems?’ and ‘Have you ever been told by a doctor that you have had a stroke?’ Participants reporting such diagnoses were classified as having CVD. The definition followed a recent 35-country analysis, confirming its feasibility for cross-national work [24].

### Independent variables

The primary independent variables, age, period, and cohort, were defined based on the characteristics specific to each survey data set: HRS, ELSA, SHARE, and CHARLS. Age was categorised into eight five-year groups: 50–54, 55–59, 60–64, 65–69, 70–74, 75–79, 80–84, and ≥85years. This consistent categorisation across all surveys allowed for comparisons across different age groups.

The period variable was defined by the specific survey years during which data were collected, reflecting distinct periods. For the HRS, periods spanned from 1992 to 2022, providing a wide temporal range. For the ELSA and SHARE extended from 2002 to 2023 and 2004 to 2021, respectively, while CHARLS covered 2011 to 2020. Each period was treated as a categorical variable corresponding to the survey years in which participants were surveyed.

Cohorts were defined according to participants’ birth years, with different categorisations reflecting the start dates of each survey. In the HRS, cohorts were categorised into thirteen 5-year birth cohort groups: pre-1910, 1910, 1915, 1920, 1925, 1930, 1935, 1940, 1945, 1950, 1955, 1960, and ≥1965. For ELSA and SHARE, cohorts were grouped into ten categories: pre-1925, 1925, 1930, 1935, 1940, 1945, 1950, 1955, 1960, and ≥1965. In CHARLS, cohorts were divided into eight groups: pre-1935, 1940, 1945, 1950, 1955, 1960, and ≥1965. These cohort groupings were specifically designed to address the identification problem in age-period-cohort analyses (*i.e*. period = age + cohort) [25], allowing us to disentangle the age, period, and cohort effects on CVD risk across different populations and periods.

### Covariates

The study controlled the following covariates: gender, marital status, educational attainment, area of residence, smoking status, and alcohol consumption during the preceding year. These covariates were treated as categorical variables and were included to account for potential confounding factors in the analysis.

### Statistical analysis

We first calculated descriptive statistics for age and period groups within each survey population, followed by the computation of the weighted prevalence of CVD. To visualise these trends, we generated plots illustrating CVD prevalence across different regions, including temporal trends by age group, generational trends by age group, and temporal trends by birth cohort.

To estimate the APC effects on CVD, we employed a hierarchical APC (H-APC) model. This model specified age as a first-level variable, while period and cohort were treated as second-level random terms. We fitted the model separately for each survey. Within each survey, the period was defined by the interview wave and specified as a random effect, allowing the model to control any wave-specific difference. Specifically, the model can be expressed as:

logit(p_ij_)=α+β_agei_+u_periodj_+v_cohortj_+X_ijγ_

where p_ij_ is the probability of CVD for individual i in period j, α is the overall intercept, β_agei_ represents the fixed effect of age, u_periodj_ denotes the random effect of period, and *v*_cohort_*_j_* denotes the random effect of cohort. The term X_ijγ_ represents the covariates and their associated effects.

Period effects u_periodj_ were modelled as random effects using a random walk of order 1 (RW1) to account for temporal dependencies:



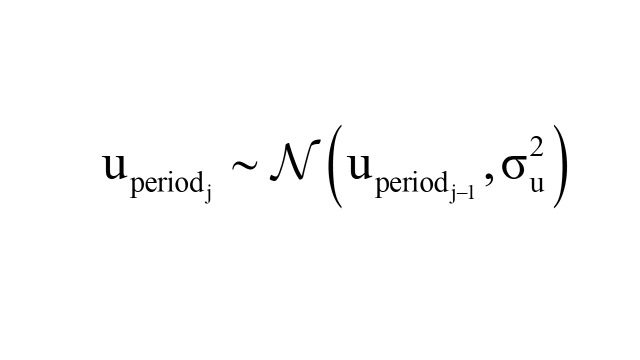



Where

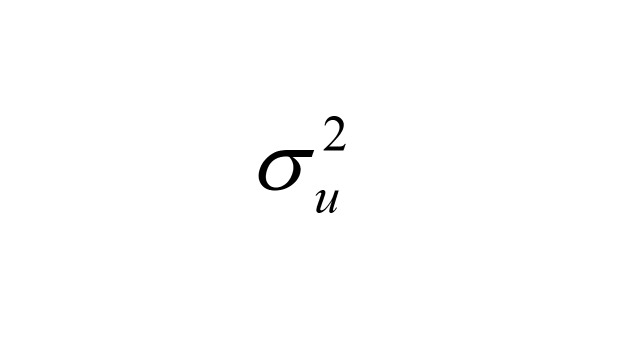



is the variance of the period effect. Cohort effects *v*_cohort_*_j_* were modelled as independent and identically distributed (IID) random effects:

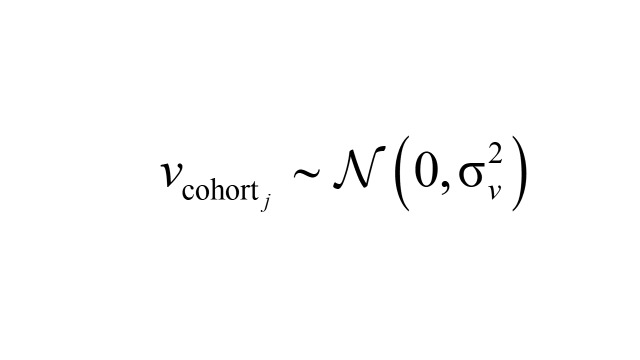



Where

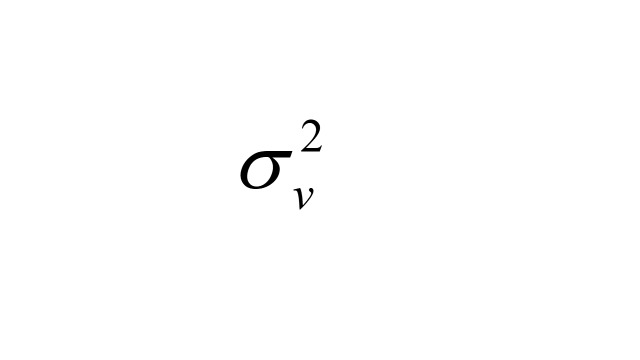



is the variance of the cohort effect. This approach is well-suited for longitudinal data, allowing for addressing the hierarchical structure and time-related dependencies inherent in the data. The model fitting was conducted using the R INLA package [26], which is well-suited for Bayesian inference in complex hierarchical models. After fitting each model, we verified numerical stability and overall fit; full diagnostics and comparison of different random effect structures are provided in the online Technical Appendix (Appendix in the **Online Supplementary Document**). Besides, we conducted sensitivity analyses in HRS and ELSA, where income data were more complete, by adding income as an additional covariate. For China, two sensitivity models were implemented: S1 restricted to respondents with recent doctor visits and S2 restricted to those taking prescribed medication, to reduce potential under-diagnosis bias.

Because age, period, and cohort are linearly dependent, the H-APC model provides descriptive rather than causal estimates. To contextualise the period effects, we conducted an ecological supplementary analysis by correlating the log of period odds ratios with four macro-level indicators linked to cardiovascular disease risk: smoking prevalence, per-capita health spending (PPP), GDP per capita (PPP), and mean exposure to fine particulate matter ≤2.5 μm in diameter (PM_2.5_). Spearman correlations were calculated for each region to assess consistency with contemporaneous macro-level trends. Subgroup analyses were subsequently conducted to explore further the APC effects across various covariates and sub-regions in Europe. We used likelihood-ratio tests (LRTs) and compared the widely applicable information criterion (WAIC) to assess whether the associations between each sociodemographic covariate and the risk of CVD varied as a moderator of period and birth cohort. To address missing data within covariates, we applied multivariate imputation by chained equations using the random forest method [27,28]. All the data analysis and visualisation were conducted in *R*, version 4.4.1 (R Foundation for Statistical Computing, Vienna, Austria).

## RESULTS

### Population characteristics

In the HRS-USA survey, participants ranged from 15 078 in 1992 to 15 491 in 2022. Similarly, in ELSA-UK, participant numbers varied from 11 515 in 2002 to 7368 in the 2021–2023 wave (**Table 1**). In the HRS-USA, the cohort born before 1910 comprised 1.30% of the total sample, with almost all members being over 85 years old by the final wave, whereas in the CHARLS-China survey, a significant portion of the population was from rural areas (61.26%), with 88.12% having less than a high school education. In contrast, Europe (SHARE-pooled countries) showed a higher proportion of participants with better educational attainment but a similar trend of increasing CVD prevalence with age, from 6.06% in younger groups to 46.27% in older groups. The UK exhibited patterns similar to the USA, with notable differences in educational attainment and urbanisation trends, reflecting the socio-economic and cultural variations between the UK and the USA (Table S1 in the **Online Supplementary Document**).

**Table 1:** Descriptive statistics by age and period groups for each survey population*

Variables		All†		50–54			55–59		60–64		65–69			70–74		75–79		80–84		≥85
		**n (%)**		**n (%)**			**n (%)**		**n (%)**		**n (%)**			**n (%)**		**n (%)**		**n (%)**		**n (%)**
**HRS-USA (n = 285 667)‡**																				
1992		15 078 (5.28)		4304 (13.61)			4339 (8.29)		2185 (4.48)		678 (1.70)			1578 (4.23)		1070 (3.40)		577 (2.46)		347 (1.66)
1994		18 529 (6.49)		2813 (8.90)			4104 (7.84)		3063 (6.28)		1105 (2.77)			2585 (6.93)		2288 (7.26)		1551 (6.62)		1020 (4.89)
1996		13 906 (4.87)		1285 (4.06)			4049 (7.73)		3709 (7.61)		1294 (3.25)			906 (2.43)		921 (2.92)		899 (3.84)		843 (4.04)
1998		20 552 (7.19)		2297 (7.26)			3785 (7.23)		3689 (7.57)		3016 (7.57)			2714 (7.28)		2282 (7.24)		1462 (6.24)		1307 (6.26)
2000		19 014 (6.66)		1622 (5.13)			2987 (5.71)		3672 (7.53)		3195 (8.02)			2452 (6.58)		2201 (6.99)		1538 (6.57)		1347 (6.45)
2002		17 747 (6.21)		783 (2.48)			2380 (4.55)		3647 (7.48)		3300 (8.28)			2572 (6.90)		2040 (6.47)		1656 (7.07)		1369 (6.56)
2004		19 259 (6.74)		2613 (8.26)			2429 (4.64)		3120 (6.40)		3395 (8.52)			2661 (7.14)		2019 (6.41)		1605 (6.85)		1417 (6.79)
2006		17 918 (6.27)		1641 (5.19)			2538 (4.85)		2357 (4.83)		3448 (8.65)			2840 (7.62)		2021 (6.41)		1555 (6.64)		1518 (7.27)
2008		16 844 (5.90)		830 (2.62)			2530 (4.83)		2153 (4.42)		3117 (7.82)			2909 (7.80)		2188 (6.94)		1525 (6.51)		1592 (7.63)
2010		21 021 (7.36)		3530 (11.16)			3668 (7.01)		2894 (5.94)		2387 (5.99)			3076 (8.25)		2341 (7.43)		1543 (6.59)		1582 (7.58)
2012		19 847 (6.95)		2266 (7.17)			3703 (7.07)		3140 (6.44)		2162 (5.43)			2912 (7.81)		2457 (7.80)		1622 (6.93)		1585 (7.59)
2014		18 267 (6.39)		1098 (3.47)			3603 (6.88)		3195 (6.55)		2297 (5.76)			2454 (6.58)		2433 (7.72)		1642 (7.01)		1545 (7.40)
2016		19 444 (6.81)		2688 (8.50)			3518 (6.72)		3270 (6.71)		2633 (6.61)			1872 (5.02)		2330 (7.39)		1666 (7.11)		1467 (7.03)
2018		17 357 (6.08)		1933 (6.11)			3567 (6.81)		2841 (5.83)		2539 (6.37)			1641 (4.40)		1916 (6.08)		1594 (6.81)		1326 (6.35)
2020		15 393 (5.39)		633 (2.00)			2866 (5.47)		2902 (5.95)		2635 (6.61)			1906 (5.11)		1607 (5.10)		1552 (6.63)		1292 (6.19)
2022		15 491 (5.42)		1286 (4.07)			2291 (4.38)		2922 (5.99)		2646 (6.64)			2201 (5.90)		1394 (4.42)		1431 (6.11)		1320 (6.32)
**ELSA-UK (n = 94 999)**																				
2002		11 515 (12.12)		2074 (19.95)			2202 (13.90)		1695 (10.11)		1713 (10.82)			1477 (10.81)		1092 (10.48)		806 (11.48)		456 (9.01)
2004		9170 (9.65)		1035 (9.95)			1890 (11.93)		1499 (8.94)		1421 (8.97)			1221 (8.94)		981 (9.41)		699 (9.96)		424 (8.38)
2006		9338 (9.83)		1625 (15.63)			1804 (11.39)		1485 (8.86)		1221 (7.71)			1150 (8.42)		919 (8.82)		625 (8.91)		509 (10.06)
2008		10 742 (11.31)		1328 (12.77)			2096 (13.23)		2141 (12.77)		1553 (9.81)			1493 (10.93)		984 (9.44)		628 (8.95)		519 (10.26)
2010		10 093 (10.62)		544 (5.23)			2016 (12.73)		2157 (12.86)		1607 (10.15)			1453 (10.64)		1080 (10.36)		678 (9.66)		558 (11.03)
2012		10 371 (10.92)		919 (8.84)			1789 (11.29)		1979 (11.80)		1868 (11.80)			1340 (9.81)		1207 (11.58)		692 (9.86)		577 (11.40)
2014		9491 (9.99)		816 (7.85)			1266 (7.99)		1818 (10.84)		1812 (11.44)			1342 (9.83)		1173 (11.25)		702 (10.00)		562 (11.11)
2016		8355 (8.79)		433 (4.16)			821 (5.18)		1623 (9.68)		1732 (10.94)			1391 (10.19)		1033 (9.91)		792 (11.29)		530 (10.47)
2018		8556 (9.01)		1080 (10.39)			807 (5.10)		1241 (7.40)		1536 (9.70)			1544 (11.31)		965 (9.26)		812 (11.57)		571 (11.28)
2021-2023		7368 (7.76)		544 (5.23)			1148 (7.25)		1129 (6.73)		1373 (8.67)			1246 (9.12)		990 (9.50)		584 (8.32)		354 (7.00)
**SHARE-pooled countries (Europe) (n = 443 723)**																				
2004		27 366 (6.17)		5041 (12.07)			5217 (7.74)		4589 (5.90)		4083 (5.29)			3369 (5.06)		2514 (4.85)		1600 (4.48)		953 (3.75)
2006		39 146 (8.82)		6147 (14.72)			7518 (11.15)		6894 (8.86)		5783 (7.49)			4943 (7.42)		3902 (7.53)		2508 (7.02)		1451 (5.70)
2011		54 747 (12.34)		7376 (17.66)			9598 (14.24)		10 215 (13.13)		8565 (11.10)			7298 (10.96)		5532 (10.68)		3764 (10.53)		2399 (9.43)
2013		64 673 (14.58)		7506 (17.97)			10 667 (15.83)		11 571 (14.87)		10 976 (14.22)			8920 (13.39)		6935 (13.39)		4796 (13.42)		3302 (12.98)
2015		66 931 (15.08)		6285 (15.05)			10 288 (15.26)		11 942 (15.35)		11 881 (15.39)			9497 (14.26)		7771 (15.00)		5333 (14.92)		3934 (15.47)
2017		75 714 (17.06)		4924 (11.79)			10 968 (16.27)		13 679 (17.58)		14 083 (18.25)			11 748 (17.64)		9192 (17.75)		6320 (17.69)		4800 (18.87)
2019		46 406 (10.46)		1035 (2.48)			4953 (7.35)		7724 (9.93)		9103 (11.79)			8467 (12.71)		6667 (12.87)		4800 (13.43)		3657 (14.38)
2021		68 740 (15.49)		3446 (8.25)			8193 (12.16)		11 193 (14.39)		12 709 (16.47)			12 362 (18.56)		9282 (17.92)		6613 (18.51)		4942 (19.43)
**CHARLS-China (n = 76 689)**																				
2011		13 281 (17.32)		2611 (16.93)			3484 (22.37)		2742 (17.41)		1792 (14.38)			1257 (15.45)		850 (16.22)		379 (13.80)		166 (12.20)
2013		14 119 (18.41)		2699 (17.50)			3285 (21.09)		3035 (19.27)		2043 (16.40)			1444 (17.75)		922 (17.60)		476 (17.33)		215 (15.80)
2015		14 770 (19.26)		3098 (20.09)			2713 (17.42)		3244 (20.60)		2364 (18.98)			1572 (19.32)		1004 (19.16)		518 (18.86)		257 (18.88)
2018		17 230 (22.47)		3543 (22.98)			2909 (18.68)		3400 (21.59)		3088 (24.79)			1919 (23.59)		1266 (24.16)		719 (26.17)		386 (28.36)
2020		17 289 (22.54)		3469 (22.50)			3185 (20.45)		3330 (21.14)		3171 (25.45)			1944 (23.89)		1198 (22.86)		655 (23.84)		337 (24.76)

*Survey Time Periods: data for the Health and Retirement Study (HRS) covers 1992–2022, the English Longitudinal Study of Ageing (ELSA) spans 2002–2023, the Survey of Health, Ageing, and Retirement in Europe (SHARE) includes 2004–2021, and the China Health and Retirement Longitudinal Study (CHARLS) covers 2011–2020.

†Age group categorisation: age groups are defined in 5-y intervals: 50–54, 55–59, 60–64, 65–69, 70–74, 75–79, 80–84, and 85+ years.

‡Sample size: sample sizes reflect individuals aged 50 y and older, excluding those with missing data for cardiovascular disease (CVD) information.

**Figure 1:**
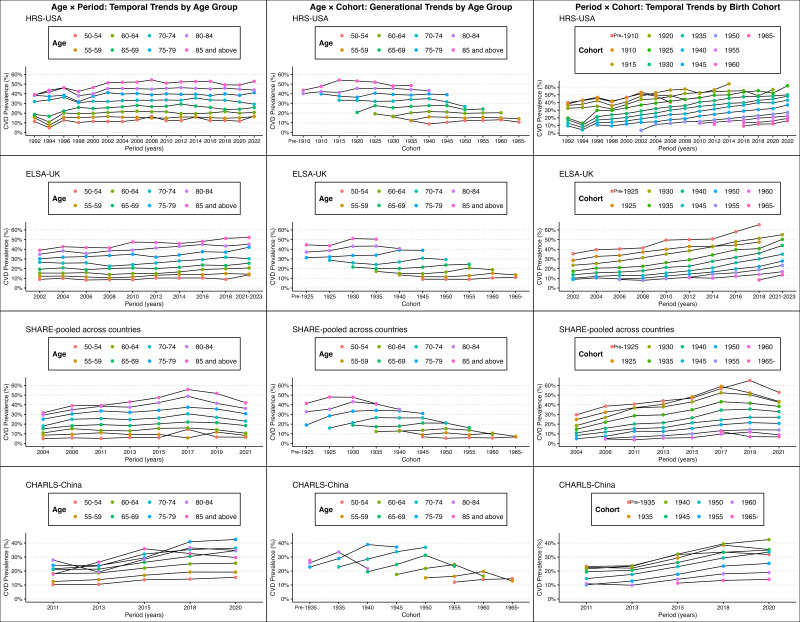
Trends plot of CVD Prevalence Across the USA, UK, Europe (SHARE-pooled countries), and China. This figure illustrates the trends in cardiovascular disease (CVD) prevalence by age group, generational trends by age group, and temporal trends by birth cohort across four regions: the USA (Health and Retirement Study, HRS), the UK (English Longitudinal Study of Ageing, ELSA), pooled European countries (Survey of Health, Ageing, and Retirement in Europe, SHARE), and China (China Health and Retirement Longitudinal Study, CHARLS). The data are weighted for each respective population. In the USA and UK, CVD prevalence remains relatively stable or increases slightly over time, with younger cohorts exhibiting lower prevalence as they age. Europe shows a notable decline in CVD prevalence, especially among older cohorts, indicating improvements in public health. In contrast, CVD prevalence is rising across all age groups in China, with younger cohorts experiencing higher rates at earlier ages, highlighting an emerging public health concern.

The trends in CVD prevalence reveal significant regional variations (**Figure 1**). In the USA and UK, CVD prevalence remains relatively stable or exhibits a modest increase over time, with a discernible generational decline indicating that younger cohorts experience lower CVD prevalence as they progress through the life course. In Europe, there has been a pronounced decrease in CVD prevalence in recent periods, particularly among older cohorts, reflecting substantial public health improvements. Conversely, China displays an increasing trend in CVD prevalence across all age groups and cohorts, with younger cohorts demonstrating higher prevalence rates at earlier ages, highlighting an emerging public health concern. These trends underscore the diverse impact of demographic shifts and public health interventions across these regions.

### Age, period, and cohort effects on the risk of CVD across different regions

**Figure 2:**
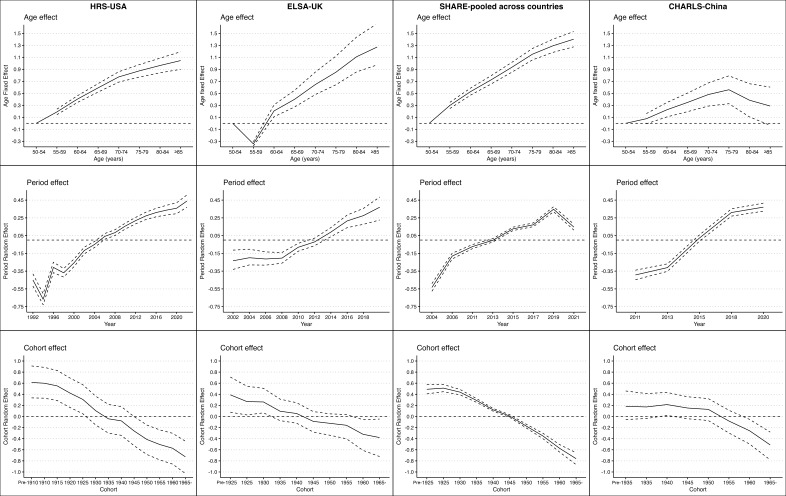
Modelled Age, Period, and Cohort Effects on CVD Risk in the USA, UK, Europe (SHARE-pooled countries), and China. This figure depicts the modelled age, period, and cohort effects on cardiovascular disease (CVD) risk in the USA, UK, SHARE-pooled European countries, and China, using Age-Period-Cohort (APC) models. The analysis controls for gender, marital status, education, residence, smoking, and alcohol consumption. The age effect shows a strong positive association with increasing age across all regions, but China shows a peak around age 75, followed by a decline. The period effect shows rising CVD risk over time in all regions, except for a recent decline in Europe. The cohort effect indicates that more recent generations have a lower CVD risk, with the most pronounced decline in the USA, UK, and Europe. In China, the decline in CVD risk is less pronounced, with a significant reduction only in the 1960- and 1965- birth cohorts.

The results are adjusted for gender, marital status, educational attainment, area of residence, living arrangement, current smoking status, and alcohol consumption in the previous year. All models converged well (**Figure 2**, **Table 2**; Appendix A in the **Online Supplementary Document**).

**Table 2:** Age, period, and cohort effects on the risk of CVD among middle-aged and older adults in the USA, UK, SHARE-pooled countries, and China

Variables	HRS-USA	ELSA-UK		SHARE-pooled countries (Europe)		CHARLS-China	
	**OR (95% CI)**	**Age**	**OR (95% CI)**	**Age**	**OR (95% CI)**	**Age**	**OR (95% CI)**
Fixed effects*							
Age							
*50–54*	1.00	50–54	1.00	50–54	1.00	50–54	1.00
*55–59*	1.21 (1.15–1.27)	55–59	0.71 (0.68–0.74)	55–59	1.35 (1.28–1.42)	55–59	1.08 (0.99–1.18)
*60–64*	1.50 (1.42–1.58)	60–64	1.23 (1.12–1.36)	60–64	1.70 (1.60–1.80)	60–64	1.25 (1.11–1.42)
*65–69*	1.81 (1.68–1.94)	65–69	1.49 (1.31–1.73)	65–69	2.06 (1.93–2.21)	65-69	1.42 (1.22–1.66)
*70–74*	2.17 (1.99–2.36)	70–74	1.91 (1.61–2.35)	70–74	2.54 (2.34–2.76)	70-74	1.61 (1.33–1.96)
*75–79*	2.40 (2.16–2.66)	75–79	2.34 (1.90–3.06)	75–79	3.16 (2.88–3.48)	75-79	1.75 (1.39–2.21)
*80–84*	2.62 (2.32–2.96)	80–84	3.03 (2.36–4.21)	80–84	3.64 (3.27–4.07)	80-84	1.47 (1.11–1.93)
*≥85*	2.85 (2.46–3.30)	≥85	3.57 (2.65–5.27)	≥85	4.06 (3.58–4.62)	≥85	1.33 (0.97–1.83)
Random effects*							
Periods							
*1992*	0.64 (0.60–0.68)						
*1994*	0.51 (0.48–0.55)						
*1996*	0.73 (0.69–0.78)						
*1998*	0.69 (0.66–0.73)						
*2000*	0.77 (0.74–0.81)						
*2002*	0.89 (0.86–0.92)	2002	0.79 (0.72–0.89)				
*2004*	0.95 (0.92–0.98)	2004	0.82 (0.76–0.90)	2004	0.59 (0.56–0.61)		
*2006*	1.04 (1.01–1.08)	2006	0.81 (0.75–0.87)	2006	0.83 (0.81–0.86)		
*2008*	1.09 (1.05–1.12)	2008	0.82 (0.77–0.87)				
*2010*	1.17 (1.13–1.21)	2010	0.92 (0.88–0.96)				
*2012*	1.24 (1.20–1.29)	2012	0.98 (0.94–1.02)	2011	0.93 (0.91–0.95)	2011	0.67 (0.64–0.71)
*2014*	1.31 (1.26–1.37)	2014	1.09 (1.04–1.15)	2013	1.00 (0.98–1.02)	2013	0.73 (0.70–0.76)
*2016*	1.36 (1.30–1.43)	2016	1.24 (1.15–1.33)	2015	1.13 (1.11–1.16)	2015	1.02 (0.99–1.06)
*2018*	1.40 (1.33–1.48)	2018	1.32 (1.19–1.43)	2017	1.19 (1.16–1.21)	2018	1.36 (1.31–1.42)
*2020*	1.43 (1.35–1.52)			2019	1.42 (1.38–1.46)	2020	1.45 (1.38–1.52)
*2022*	1.55 (1.45–1.66)	2021-2023	1.45 (1.25–1.62)	2021	1.16 (1.12–1.20)		
Cohort							
*pre-1910*	1.85 (1.40–2.49)						
*1910*	1.82 (1.39–2.42)						
*1915*	1.74 (1.33–2.29)						
*1920*	1.53 (1.18–2.00)	pre -1925	1.48 (1.08–2.04)	pre -1925	1.63 (1.50–1.77)		
*1925*	1.36 (1.05–1.77)	1925	1.31 (1.03–1.73)	1925	1.67 (1.56–1.78)		
*1930*	1.11 (0.86–1.44)	1930	1.30 (1.07–1.66)	1930	1.55 (1.48–1.63)	pre -1935	1.20 (0.95–1.58)
*1935*	0.96 (0.74–1.24)	1935	1.10 (0.93–1.37)	1935	1.33 (1.29–1.38)	1935	1.19 (0.96–1.51)
*1940*	0.92 (0.71–1.20)	1940	1.06 (0.89–1.28)	1940	1.13 (1.10–1.16)	1940	1.24 (1.02–1.54)
*1945*	0.77 (0.59–1.00)	1945	0.92 (0.76–1.09)	1945	1.01 (0.98–1.03)	1945	1.16 (0.96–1.43)
*1950*	0.66 (0.50–0.86)	1950	0.88 (0.71–1.05)	1950	0.84 (0.81–0.87)	1950	1.14 (0.93–1.38)
*1955*	0.60 (0.46–0.79)	1955	0.85 (0.67–1.04)	1955	0.70 (0.67–0.74)	1955	0.92 (0.74–1.12)
*1960*	0.56 (0.42–0.74)	1960	0.73 (0.54–0.94)	1960	0.56 (0.53–0.60)	1960	0.77 (0.61–0.95)
*≥1965*	0.48 (0.36 ~ 0.64)	≥1965	0.68 (0.49–0.95)	≥1965	0.47 (0.42–0.52)	≥1965	0.60 (0.46–0.75)

CI – confidence interval, CVD – cardiovascular diseases, OR – odds ratio

*Values represent OR (95% CI) for fixed effects and random effects. All the models were adjusted for gender, marital status, educational attainment, area of residence, living, current smoking status, and alcohol consumption last year.

The results reveal a consistent increase in the risk of CVD with advancing age across all four regions. In the USA, the odds ratio (OR) for CVD increases steadily from 1.21 (95% CI = 1.15–1.27) for those aged 55–59 years to 2.85 (95% CI = 2.46–3.30) for those aged 85 and older. This trend is also observed in the UK, where the OR rises from 0.71 (95% CI = 0.68–0.74) in the 55–59 age group to 3.57 (95% CI = 2.65–5.27) in the ≥85 age group. In Europe and CHARLS, the CVD risk follows a comparable upward trajectory with age, although the magnitude of risk varies slightly between regions. Moreover, the age effects in China peak around age 75 (OR = 1.75; 95% CI = 1.39 –2.21), followed by a decline at age 85 and more (OR = 1.33; 95% CI = 0.97–1.83). Adding income in HRS and ELSA did not change age, period, cohort, or subgroup patterns (Table S2–3 in the **Online Supplementary Document**). In China, age-specific odds ratios after age 75 rose in S1 and S2, and the decline became a plateau, though a slight dip remained after age 80 (Table S4 in the **Online Supplementary Document**).

The period effects, which reflect changes in CVD risk over time, vary significantly across the regions. In the USA, there is a gradual increase in CVD risk over time, with the OR rising from 0.64 (95% CI = 0.60–0.68) in 1992 to 1.55 (95% CI = 1.45–1.66) in 2022, suggesting an overall worsening of CVD risk over the study period. The UK shows a more stable period effect, slightly increasing over time, from an OR of 0.79 (95% CI = 0.72–0.89) in 2002 to 1.45 (95% CI = 1.25–1.62) in 2021–2023. In Europe, the period effect is somewhat mixed, with early years showing lower risk (OR = 0.59 in 2004) and later years showing a higher risk (OR = 1.42 in 2019). While China exhibits a notable increase in CVD risk over time, with a significant rise from an OR = 0.67 (95% CI = 0.64–0.71) in 2011 to 2020 (OR = 1.45; 95% CI = 1.38–1.52), indicating a growing public health challenge.

Regarding the cohort effect, the results reveal a declining risk of CVD in more recent birth cohorts across all regions. In the USA, the OR for the risk of CVD decreases from 1.85 (95% CI = 1.40–2.49) in the pre-1910 cohort to 0.48 (95% CI = 0.36–0.64) in the ≥1965 cohort. Similarly, in the UK, the OR declines from 1.48 (95% CI = 1.08–2.04) in the pre-1925 cohort to 0.68 (95% CI = 0.49–0.95) in the ≥1965 cohort. Europe and China also exhibit similar downward trends, though the specific magnitude differs, with China showing a less pronounced decline. In China, only the 1960 and 1965 cohorts have a significantly lower CVD risk with OR of 0.77 (95% CI = 0.61–0.95) and 0.60 (95% CI = 0.46–0.75).

We also found the period log(OR) was correlated with smoking prevalence, GDP, health spending, and PM_2.5_ exposure (Figure S1 in the **Online Supplementary Document**). These associations indicate that the estimated period effects are consistent with well-documented macro-level changes in risk factors during the study period.

### Sociodemographic and regional variations in CVD risk: APC model insights

**Figure 3:**
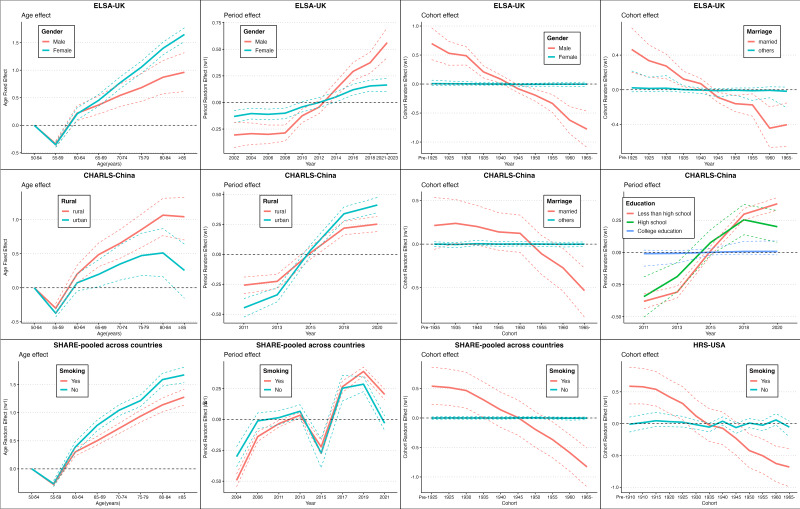
Modelled Age, Period, and Cohort Effects on CVD Risk Stratified by Sociodemographic Groups in the USA, UK, SHARE-pooled countries, and China. This figure presents the age, period, and cohort effects on cardiovascular disease (CVD) risk across the USA, UK, SHARE-pooled European countries, and China, stratified by significant sociodemographic factors. Only statistically significant interactions, identified using likelihood-ratio tests (*P* < 0.05) and widely applicable information criterion (WAIC) (ΔWAIC>2), are displayed. Full subgroup results are provided in the **Online Supplementary Document**. The analysis reveals the age, period, and cohort effects on CVD risk, stratified by sociodemographic factors, including gender, marital status, education, smoking, and urban/rural residence. In general, CVD risk increases with age across all regions, with higher risks among females, rural residents, and non-smokers. Period effects show rising CVD risk over time, particularly among smokers, urban residents, and individuals with lower education. Cohort effects reveal a decline in CVD risk among married individuals in the UK and China, males in the UK, and smokers in the UK and the USA in more recent cohorts, highlighting the impact of public health interventions and lifestyle changes.

We further evaluated whether the associations between age, period, birth cohort effect, and CVD risks vary depending on each sociodemographic covariate (Figure S2–7 in the **Online Supplementary Document**). We highlight only the significant sociodemographic moderators of CVD risk, focusing on key factors such as gender, education, marital status, and more across the USA, UK, Europe, and China (**Figure 3**).

The analysis reveals that CVD risk consistently increased with age across all regions studied. Notably, certain demographic groups, including females in the UK, rural residents in China, and non-smokers in Europe, exhibit higher risks. Period effects indicate a general increase in CVD risk over time, with this trend being particularly evident among males in the UK, smokers in Europe, urban residents, and individuals with lower educational attainment in China. Conversely, cohort effects show a decline in CVD risk among subgroups in more recent birth cohorts. This decline is especially noticeable among married individuals in the UK and China, males in the UK, and smokers in the UK and the USA.

We also found consistent regional trends in CVD risk across Northern, Western, Southern, and Eastern Europe. The results show that CVD risk generally increases with age and highlight a period effect that peaks around 2017–2019, followed by stabilisation or decline, particularly in Northern and Western Europe (Figure S8 in the **Online Supplementary Document**). These trends suggest improvements in CVD management and health care delivery in these regions.

## DISCUSSION

In this study, we aimed to investigate the factors underlying the increasing trends in CVD by employing a comprehensive age-period-cohort analysis across four regions: the USA, the UK, Europe, and China. Our findings reveal a consistent increase in CVD risk with advancing age across all regions, highlighting the well-established relationship between ageing and cardiovascular health. Moreover, the period effects indicate an increase in CVD risk over time in the USA and China, in contrast with more stable or improving trends in Europe. Additionally, the cohort effects reveal a declining risk of CVD in more recent birth cohorts across all regions, although the magnitude of this decline varies, with China showing a less pronounced reduction. Moreover, we observed substantial cross-country variations and sociodemographic disparities in these trends, emphasising the complex interplay of demographic, temporal, and socioeconomic factors shaping global CVD patterns. These findings contribute to the growing body of evidence on global CVD burden and emphasise the need for region-specific public health strategies to mitigate the burden of CVD as populations age.

This study demonstrates a consistent increase in CVD risk with increased age across all regions, reflecting the well-established relationship between ageing and cardiovascular health deterioration [3]. In most regions, this increase in risk persists steadily, with the highest CVD risk observed in the oldest age groups. However, we also found a distinct pattern in China, where CVD risk peaks around age 75 and then declines among those aged 85 and older. Sensitivity analyses (Table S4 in the **Online Supplementary Document**) showed that the decline weakened after restricting to respondents with recent care or medication use, indicating that underdiagnosis explains much of the pattern. A slight drop after age 80 remained, likely due to selective survival among healthier individuals. A previous study in China also found similar evidence that a functional disability decline among the oldest-old group (*i.e*. aged ≥80 years) was partly due to mortality and loss of follow-up [29]. Despite these regional differences, the overall trend highlights the impact of ageing on cardiovascular health, emphasising the need for age-specific preventive measures and interventions across all populations [3].

The period effects estimated by the H-APC model show variations across regions in CVD risk. In the USA, UK, and China, the period effects indicate a rising trend in CVD risk over recent decades. The trend is consistent with the growing influence of contemporary lifestyle factors, environmental changes, and health care system shifts [12,13], but these associations are ecological rather than causal. In contrast, Europe has experienced a recent decline in CVD risk. The period effect in Europe indicates a peak in CVD risk around 2017–2019, followed by stabilisation or decline during 2019–2021, particularly in Northern and Western Europe. This finding is consistent with previous evidence, which also found a decline in CVD mortality in Northern and Western Europe, likely associated with improved health care infrastructure, public health interventions, and better management of risk factors like smoking and air pollution. However, it also found that the burden remains high, where preventive measures and health care improvement have been slower [30].

The cohort effects highlight the significant improvement in CVD risk among younger generations across the studied regions. In the USA, UK, and Europe, there is an apparent decline in CVD risk among more recent cohorts, indicating successful public health measures and improved early-life conditions. In China, however, only the 1960 and 1965 cohorts experienced a measurable reduction. Earlier Chinese cohorts do not benefit, which is consistent with evidence on early-life adversity [8]. These findings implied a potential exposure window during this birth cohort for older Chinese adults and emphasised the need for continued focus on cohort-specific interventions, particularly in regions where progress has been slower [5].

Sociodemographic factors reveal distinct CVD risk patterns across regions. While age remains a consistent risk factor, certain groups, such as females in the UK, rural residents in China, and non-smokers in Europe, show higher risks. In the UK, the elevated risk among females may be linked to hormonal changes associated with menopause [17]. Rural residents in China are at higher risk due to health care disparities [14,31], while non-smokers in Europe, including former smokers, face increased risk due to past smoking [32]. Period effects show rising CVD risk over time, particularly among males in the UK, smokers in Europe, and urban residents with lower education in China, driven by factors like sedentary behaviour and obesity [4,16]. The protective effect observed in married individuals could be attributed to the psychosocial benefits of marriage, such as emotional support and shared financial resources, which are known to enhance health outcomes [33].

In Northern and Western Europe, the recent decline in CVD risk suggests that these regions are further in the health transition, effectively managing traditional risk factors through comprehensive public health policies, advanced health care systems, and widespread adoption of healthier lifestyles [30]. These successes align with the World Health Organization’s (WHO) emphasis on healthy ageing, supporting longer, healthier lives for ageing populations. However, in the USA and the UK, rising CVD risks highlight the ongoing challenges in addressing modern lifestyle-related factors such as obesity and smoking [12]. Despite active public health efforts, these countries face a dual burden of managing traditional and emerging cardiovascular risks. This situation underscores the need for innovative public health strategies that are adaptable to the evolving needs of their populations, ensuring alignment with the WHO’s goal of promoting healthy ageing globally [34].

China is undergoing rapid urbanisation and lifestyle change that push CVD risk higher across all ages [13]. In contrast to Europe and North America, China lags in this transition, struggling with the surge in risk factors without a fully established public health system to mitigate their impact [35]. Aligning these efforts with the WHO’s vision of healthy ageing is critical to ensure that all segments of China’s population can experience longevity with a high quality of life. Population-level strategies, primary care strengthening, and tertiary care reforms are urgently needed [35].

Our findings have implications for the WHO’s focus on reducing non-communicable diseases and promoting healthy ageing [2]. The increase in CVD risk with age across regions, along with the rise in risk over time in the USA, UK, and China, reflects the growing global burden of CVD, particularly in ageing populations. These trends emphasise the need for targeted, region-specific public health strategies to address the diverse sociodemographic factors influencing CVD risk, such as gender, education, and rural *vs*. urban residence [36]. Although our APC models use person-level data, the estimated age, period, and cohort effects describe average patterns for each country and may not apply equally to all subgroups; policy decisions should therefore be informed by local data on regional, socioeconomic, and ethnic variation. WHO’s emphasis on integrated health services is echoed in our findings, as integrating cardiovascular care with other health services, such as mental health and primary care, is crucial to mitigating risk and ensuring equitable access to care [37]. Additionally, the observed sociodemographic disparities highlight the importance of addressing health inequalities, particularly in vulnerable groups, to support the WHO’s goal of promoting healthier ageing globally [38].

### Strengths and limitations

This study has several strengths. First, nationally representative data sets from multiple countries enable a comprehensive cross-country comparative analysis, providing critical insights into CVD risks across diverse socio-economic and health care environments. Second, by extending the analysis to include middle-aged individuals (starting at age 50), the study captures the early onset and progression of CVD risk factors, thereby broadening the scope of its relevance to a broader demographic. Third, the application of APC analysis is particularly noteworthy, as it allows for a detailed examination of the relative contributions of early-life exposures, contemporaneous factors, and age-related processes to CVD risk, thereby offering a nuanced understanding of the interplay between these elements over time. Additionally, using Bayesian inference, specifically through the integrated nested Laplace approximation, represents a methodological strength, providing an efficient and precise framework for implementing smoothing models. This approach significantly enhances the computational efficiency and accuracy of the analysis, particularly in the context of complex hierarchical data.

Despite its strengths, this study has several limitations that should be acknowledged. First, the reliance on self-reported disease events introduces the possibility of misclassification and underreporting, primarily through underreporting. While some true cases may be missed due to recall bias or limited diagnostic access, validation studies suggest that false positives are limited [39,40]. This pattern of error would likely lead to conservative estimates of CVD prevalence but is unlikely to distort the age, period, or cohort trends observed within each survey. Second, the descriptive nature of this study limits its ability to establish causal relationships; thus, the interpretations of our findings remain speculative and should be approached with caution. Third, despite the use of harmonised data sets, differences in survey design, cultural interpretations of illness, and definitions of socioeconomic variables across countries may introduce residual inconsistencies in cross-national comparisons. Additionally, excluding specific samples due to missing data introduces the risk of selection bias, which could influence the generalisability of the results. Although we employed multiple imputations using random forest methods to address missing data, this robust approach may not fully eliminate the potential biases introduced by excluding incomplete cases.

## CONCLUSIONS

In conclusion, this study found the dynamics of CVD trends across global regions, highlighting the roles of age, period, cohort effects, and sociodemographic moderators. Our findings highlight the importance of identifying vulnerable populations and guiding public health interventions to promote healthy ageing and reduce the global burden of CVD. Life course interventions tailored to high-risk birth cohorts or periods could play a key role in mitigating health disparities and improving outcomes. Moreover, the results emphasise the need for region-specific strategies to address the unique challenges faced by different populations, particularly in rapidly developing countries. Finally, further research is essential to better understand the interactions between age-period-cohort effects and socio-economic factors across diverse contexts, which will be crucial for developing more effective public health strategies for CVD worldwide.

## Additional material


Online Supplementary Document
