# Supplementary material for: Age-period-cohort analysis of cardiovascular disease trends in middle-aged and older adults: cross-country comparison across HRS, ELSA, SHARE, and CHARLS
Source: J Glob Health. 2025 Sep 12;15:04260. doi: 10.7189/jogh.15.04260 (PMC12434385; doi:10.7189/jogh.15.04260)
Supplement: Online Supplementary Document [file jogh-15-04260-s001.pdf]

Supplement to:

Li J, Lin S, Pei H, Xie G, Pei L, Chen G. Age-period-cohort analysis of cardiovascular disease trends in middle-aged and older adults: cross-country comparison across HRS, ELSA, SHARE, and CHARLS. J Glob Health. 2025;15:04260.

Supplementary Table of Contents

Tables

Supplementary Table S1. Descriptive Statistics by Age and Cohort Group and Other Demographic Variables for Each Survey Population
Supplementary Table S2. Sensitivity Analysis of Age, Period, and Cohort Effects on CVD Risk among Middle-Aged and Older Adults in the USA and UK (Income-Adjusted Models)
Supplementary Table S3. Model Diagnostics for Sensitivity Analysis (Income-Adjusted HRS and ELSA Models) Compared with Main Models
Supplementary Table S4. CHARLS Sensitivity Analysis of Late-Life APC Effects under Alternative Health-Care-Contact Restrictions

Figures

Supplementary Figure S1. Correlation Between Modelled Period Effects and Macro-Level Indicators in the USA, UK, SHARE-Pooled Countries, and China
Supplementary Figure S2. Modelled Age, Period, and Cohort Effects on CVD Risk Stratified by Gender in the USA, UK, SHARE-Pooled Countries, and China
Supplementary Figure S3. Modelled Age, Period, and Cohort Effects on CVD Risk Stratified by Marriage Status in the USA, UK, SHARE-Pooled Countries, and China.
Supplementary Figure S4. Modelled Age, Period, and Cohort Effects on CVD Risk Stratified by Education in the USA, UK, SHARE-Pooled Countries, and China.
Supplementary Figure S5. Modelled Age, Period, and Cohort Effects on CVD Risk Stratified by Rural Residence in the USA, UK, SHARE-Pooled Countries, and China.
Supplementary Figure S6. Modelled Age, Period, and Cohort Effects on CVD Risk Stratified by Smoking Status in the USA, UK, SHARE-Pooled Countries, and China.
Supplementary Figure S7. Modelled Age, Period, and Cohort Effects on CVD Risk Stratified by Drinking Status in the USA, UK, SHARE-Pooled Countries, and China.
Supplementary Figure S8. Modelled Age, Period, and Cohort Effects on CVD Risk Across Northern, Western, Southern, and Eastern Europe

Online Technical Appendix A – Model specification, priors, diagnostics, and robustness

A-1 Hierarchical age–period–cohort (H-APC) likelihood and identifiability
A-2 Prior specification and rationale (non-informative scheme)
A-3 Convergence and overall model fit
A-4 Sensitivity to prior precision
A-5 Sensitivity to alternative period- and cohort-correlation structures

Supplementary Table S1. Descriptive Statistics by Age and Cohort Group and Other Demographic Variables for Each Survey Population

Variables	All	50-54	55-59	60-64	65-69	70-74	75-79	80-84	≥85
	n(%)	n(%)	n(%)	n(%)	n(%)	n(%)	n(%)	n(%)	n(%)
**HRS-USA(N=283,107)**									
**Cohort**									
-1910	3,721 (1.30)	0 (0.00)	0 (0.00)	0 (0.00)	0 (0.00)	0 (0.00)	0 (0.00)	221 (0.94)	3,500 (16.76)
1910	6,712 (2.35)	0 (0.00)	0 (0.00)	0 (0.00)	0 (0.00)	0 (0.00)	352 (1.12)	2,910 (12.43)	3,450 (16.53)
1915	11,800 (4.13)	0 (0.00)	0 (0.00)	0 (0.00)	0 (0.00)	541 (1.45)	4,070 (12.92)	3,560 (15.20)	3,629 (17.38)
1920	18,509 (6.48)	0 (0.00)	0 (0.00)	0 (0.00)	308 (0.77)	5,064 (13.58)	5,240 (16.63)	3,998 (17.07)	3,899 (18.68)
1925	21,227 (7.43)	0 (0.00)	0 (0.00)	549 (1.13)	2,823 (7.08)	5,809 (15.58)	5,066 (16.08)	3,703 (15.81)	3,277 (15.70)
1930	36,951 (12.93)	0 (0.00)	2,258 (4.31)	8,221 (16.86)	7,716 (19.36)	6,757 (18.13)	5,444 (17.28)	4,142 (17.69)	2,413 (11.56)
1935	48,220 (16.88)	2,481 (7.85)	10,088 (19.27)	9,333 (19.14)	8,493 (21.31)	7,168 (19.23)	6,119 (19.42)	3,834 (16.37)	704 (3.37)
1940	39,702 (13.90)	6,414 (20.28)	8,117 (15.50)	7,319 (15.01)	6,511 (16.34)	6,035 (16.19)	4,259 (13.52)	1,045 (4.46)	2 (0.01)
1945	27,499 (9.63)	4,131 (13.06)	6,099 (11.65)	6,210 (12.74)	5,801 (14.56)	4,298 (11.53)	957 (3.04)	2 (0.01)	1 (0.00)
1950	27,788 (9.73)	4,555 (14.40)	7,471 (14.27)	7,897 (16.20)	6,255 (15.70)	1,607 (4.31)	1 (0.00)	1 (0.00)	1 (0.00)
1955	24,059 (8.42)	5,966 (18.87)	8,968 (17.13)	7,185 (14.74)	1,938 (4.86)	0 (0.00)	0 (0.00)	1 (0.00)	1 (0.00)
1960	14,235 (4.98)	4,363 (13.80)	7,829 (14.95)	2,040 (4.18)	2 (0.01)	0 (0.00)	0 (0.00)	1 (0.00)	0 (0.00)
1965-	5,244 (1.84)	3,712 (11.74)	1,527 (2.92)	5 (0.01)	0 (0.00)	0 (0.00)	0 (0.00)	0 (0.00)	0 (0.00)
**Gender**									

Male	121,043 (42.37)	12,178 (38.51)	22,986 (43.90)	21,689 (44.48)	17,831 (44.75)	16,376 (43.93)	13,457 (42.71)	9,434 (40.29)	7,092 (33.97)
Female	164,624 (57.63)	19,444 (61.49)	29,371 (56.10)	27,070 (55.52)	22,016 (55.25)	20,903 (56.07)	18,051 (57.29)	13,984 (59.71)	13,785 (66.03)
**Education**									
Less than high school	65,206 (23.04)	5,153 (16.82)	9,535 (18.44)	10,010 (20.66)	8,682 (21.95)	9,078 (24.49)	8,506 (27.11)	7,017 (30.06)	7,225 (34.69)
High school or vocational education	159,871 (56.50)	18,394 (60.03)	30,335 (58.66)	28,040 (57.88)	22,362 (56.53)	20,773 (56.04)	17,210 (54.85)	12,410 (53.16)	10,347 (49.68)
College education	57,897 (20.46)	7,093 (23.15)	11,844 (22.90)	10,397 (21.46)	8,515 (21.52)	7,216 (19.47)	5,658 (18.03)	3,917 (16.78)	3,257 (15.64)
Missing	2,693	982	643	312	288	212	134	74	48
**Marriage**									
married	170,454 (59.72)	21,747 (68.83)	34,026 (65.08)	31,854 (65.40)	26,335 (66.14)	23,293 (62.54)	17,358 (55.12)	10,385 (44.38)	5,456 (26.16)
Others	114,950 (40.28)	9,848 (31.17)	18,261 (34.92)	16,854 (34.60)	13,483 (33.86)	13,951 (37.46)	14,133 (44.88)	13,017 (55.62)	15,403 (73.84)
Missing	263	27	70	51	29	35	17	16	18
**Rural area**									
Urban	199,458 (72.30)	22,404 (74.53)	37,396 (74.07)	34,245 (72.65)	27,177 (70.66)	25,605 (70.72)	21,789 (71.10)	16,303 (71.79)	14,539 (72.08)
rural	76,414 (27.70)	7,657 (25.47)	13,088 (25.93)	12,889 (27.35)	11,286 (29.34)	10,599 (29.28)	8,858 (28.90)	6,406 (28.21)	5,631 (27.92)
Missing	9,795	1,561	1,873	1,625	1,384	1,075	861	709	707
**Smoking**									
No	242,330 (85.28)	23,757 (75.37)	40,574 (77.94)	39,559 (81.57)	34,045 (85.90)	33,088 (89.24)	28,937 (92.32)	22,081 (94.85)	20,289 (97.72)
Yes	41,842 (14.72)	7,765 (24.63)	11,485 (22.06)	8,936 (18.43)	5,588 (14.10)	3,988 (10.76)	2,407 (7.68)	1,199 (5.15)	474 (2.28)
Missing	1,495	100	298	264	214	203	164	138	114
**Drinking**									

No	138,081 (48.35)	11,649 (36.85)	21,274 (40.65)	21,866 (44.85)	18,996 (47.68)	18,984 (50.94)	17,485 (55.50)	13,992 (59.78)	13,835 (66.29)
Yes	147,508 (51.65)	19,960 (63.15)	31,067 (59.35)	26,885 (55.15)	20,843 (52.32)	18,286 (49.06)	14,019 (44.50)	9,414 (40.22)	7,034 (33.71)
Missing	78	13	16	8	8	9	4	12	8
**CVD Status**									
No	207,018 (72.47)	27,914 (88.27)	44,184 (84.39)	38,654 (79.28)	29,188 (73.25)	24,808 (66.55)	19,149 (60.78)	12,937 (55.24)	10,184 (48.78)
Yes	78,649 (27.53)	3,708 (11.73)	8,173 (15.61)	10,105 (20.72)	10,659 (26.75)	12,471 (33.45)	12,359 (39.22)	10,481 (44.76)	10,693 (51.22)
**ELSA-UK(N=94,999)**									
**Cohort**									
-1925	5,528 (5.82)	0 (0.00)	0 (0.00)	0 (0.00)	0 (0.00)	0 (0.00)	496 (4.76)	1,977 (28.17)	3,055 (60.38)
1925	5,902 (6.21)	0 (0.00)	0 (0.00)	0 (0.00)	0 (0.00)	730 (5.35)	2,373 (22.76)	1,637 (23.33)	1,162 (22.96)
1930	8,668 (9.12)	0 (0.00)	0 (0.00)	0 (0.00)	813 (5.13)	2,984 (21.85)	2,460 (23.60)	1,789 (25.49)	622 (12.29)
1935	12,130 (12.77)	0 (0.00)	0 (0.00)	804 (4.80)	3,432 (21.67)	3,450 (25.26)	2,918 (27.99)	1,305 (18.60)	221 (4.37)
1940	13,771 (14.50)	0 (0.00)	999 (6.31)	3,689 (22.00)	3,747 (23.66)	3,408 (24.95)	1,618 (15.52)	310 (4.42)	0 (0.00)
1945	18,504 (19.48)	1,123 (10.80)	4,686 (29.59)	5,049 (30.11)	4,614 (29.14)	2,473 (18.11)	559 (5.36)	0 (0.00)	0 (0.00)
1950	16,193 (17.05)	3,532 (33.97)	4,883 (30.83)	4,611 (27.50)	2,558 (16.15)	609 (4.46)	0 (0.00)	0 (0.00)	0 (0.00)
1955	8,326 (8.76)	2,410 (23.18)	3,174 (20.04)	2,069 (12.34)	671 (4.24)	2 (0.01)	0 (0.00)	0 (0.00)	0 (0.00)
1960	3,976 (4.19)	1,907 (18.34)	1,522 (9.61)	545 (3.25)	1 (0.01)	1 (0.01)	0 (0.00)	0 (0.00)	0 (0.00)
1965-	2,001 (2.11)	1,426 (13.71)	575 (3.63)	0 (0.00)	0 (0.00)	0 (0.00)	0 (0.00)	0 (0.00)	0 (0.00)
**Gender**									

Male	42,671 (44.92)	4,219 (40.58)	7,098 (44.81)	7,759 (46.28)	7,458 (47.10)	6,439 (47.15)	4,774 (45.80)	2,987 (42.56)	1,937 (38.28)
Female	52,328 (55.08)	6,179 (59.42)	8,741 (55.19)	9,008 (53.72)	8,378 (52.90)	7,218 (52.85)	5,650 (54.20)	4,031 (57.44)	3,123 (61.72)
**Education**									
Less than high school	30,797 (34.32)	1,988 (20.37)	3,533 (23.45)	4,446 (27.66)	5,059 (33.47)	5,173 (40.08)	4,512 (46.35)	3,375 (52.29)	2,711 (58.59)
High school or vocational education	42,493 (47.35)	5,439 (55.72)	8,070 (53.56)	8,114 (50.49)	7,193 (47.59)	5,788 (44.84)	3,974 (40.83)	2,403 (37.23)	1,512 (32.68)
College education	16,449 (18.33)	2,334 (23.91)	3,465 (23.00)	3,511 (21.85)	2,864 (18.95)	1,946 (15.08)	1,248 (12.82)	677 (10.49)	404 (8.73)
Missing	5,260	637	771	696	720	750	690	563	433
**Marriage**									
married	63,234 (66.58)	7,532 (72.44)	11,504 (72.65)	12,408 (74.02)	11,361 (71.76)	9,221 (67.52)	6,300 (60.44)	3,402 (48.48)	1,506 (29.77)
Others	31,747 (33.42)	2,865 (27.56)	4,331 (27.35)	4,355 (25.98)	4,470 (28.24)	4,435 (32.48)	4,123 (39.56)	3,616 (51.52)	3,552 (70.23)
Missing	18	1	4	4	5	1	1	0	2
**Smoking**									
No	82,182 (87.15)	8,239 (79.76)	12,835 (81.76)	14,141 (84.96)	13,876 (88.12)	12,221 (90.03)	9,507 (91.77)	6,545 (94.02)	4,818 (96.69)
Yes	12,116 (12.85)	2,091 (20.24)	2,863 (18.24)	2,503 (15.04)	1,871 (11.88)	1,354 (9.97)	853 (8.23)	416 (5.98)	165 (3.31)
Missing	701	68	141	123	89	82	64	57	77
**Drinking**									
No	11,347 (13.71)	922 (10.31)	1,441 (10.32)	1,671 (11.18)	1,775 (12.38)	1,769 (14.44)	1,624 (17.89)	1,241 (21.60)	904 (25.81)
Yes	71,417 (86.29)	8,021 (89.69)	12,517 (89.68)	13,275 (88.82)	12,565 (87.62)	10,483 (85.56)	7,453 (82.11)	4,505 (78.40)	2,598 (74.19)
Missing	12,235	1,455	1,881	1,821	1,496	1,405	1,347	1,272	1,558
**CVD Status**									

No	73,475 (77.34)	9,387 (90.28)	13,866 (87.54)	14,074 (83.94)	12,481 (78.81)	9,989 (73.14)	6,833 (65.55)	4,149 (59.12)	2,696 (53.28)
Yes	21,524 (22.66)	1,011 (9.72)	1,973 (12.46)	2,693 (16.06)	3,355 (21.19)	3,668 (26.86)	3,591 (34.45)	2,869 (40.88)	2,364 (46.72)
**SHARE-pooled countries (Europe) (N=443,723)**									
**Cohort**									
-1925	9,991 (2.25)	0 (0.00)	0 (0.00)	0 (0.00)	0 (0.00)	0 (0.00)	92 (0.18)	2,653 (7.42)	7,246 (28.48)
1925	18,193 (4.10)	0 (0.00)	0 (0.00)	0 (0.00)	0 (0.00)	157 (0.24)	4,149 (8.01)	4,991 (13.97)	8,896 (34.97)
1930	33,140 (7.47)	0 (0.00)	0 (0.00)	0 (0.00)	194 (0.25)	5,432 (8.16)	7,739 (14.94)	12,455 (34.85)	7,320 (28.78)
1935	49,320 (11.12)	0 (0.00)	0 (0.00)	260 (0.33)	6,715 (8.70)	10,095 (15.16)	18,049 (34.85)	12,225 (34.21)	1,976 (7.77)
1940	63,095 (14.22)	0 (0.00)	238 (0.35)	7,518 (9.66)	11,881 (15.39)	22,961 (34.47)	17,088 (32.99)	3,409 (9.54)	0 (0.00)
1945	78,166 (17.62)	233 (0.56)	8,693 (12.90)	14,283 (18.36)	28,234 (36.58)	22,044 (33.10)	4,678 (9.03)	1 (0.00)	0 (0.00)
1950	81,521 (18.37)	8,332 (19.95)	13,973 (20.73)	29,003 (37.28)	24,299 (31.48)	5,914 (8.88)	0 (0.00)	0 (0.00)	0 (0.00)
1955	63,587 (14.33)	10,910 (26.13)	25,239 (37.45)	21,577 (27.73)	5,860 (7.59)	1 (0.00)	0 (0.00)	0 (0.00)	0 (0.00)
1960	37,317 (8.41)	16,134 (38.64)	16,018 (23.76)	5,165 (6.64)	0 (0.00)	0 (0.00)	0 (0.00)	0 (0.00)	0 (0.00)
1965-	9,393 (2.12)	6,151 (14.73)	3,241 (4.81)	1 (0.00)	0 (0.00)	0 (0.00)	0 (0.00)	0 (0.00)	0 (0.00)
**Gender**									
Male	194,492 (43.83)	16,603 (39.76)	29,043 (43.09)	34,892 (44.84)	35,452 (45.93)	30,505 (45.80)	23,054 (44.51)	15,126 (42.33)	9,817 (38.59)
Female	249,231 (56.17)	25,157 (60.24)	38,359 (56.91)	42,915 (55.16)	41,731 (54.07)	36,099 (54.20)	28,741 (55.49)	20,608 (57.67)	15,621 (61.41)
**Education**									

Less than high school	176,276 (39.73)	11,160 (26.73)	19,853 (29.46)	25,725 (33.07)	28,775 (37.29)	28,421 (42.67)	25,892 (49.99)	20,110 (56.28)	16,340 (64.23)
High school or vocational education	170,977 (38.53)	19,097 (45.73)	30,225 (44.85)	33,737 (43.36)	31,236 (40.47)	24,593 (36.93)	16,430 (31.72)	9,918 (27.76)	5,741 (22.57)
College education	96,440 (21.74)	11,500 (27.54)	17,317 (25.69)	18,337 (23.57)	17,164 (22.24)	13,588 (20.40)	9,471 (18.29)	5,706 (15.97)	3,357 (13.20)
Missing	30	3	7	8	8	2	2	0	0
**Marriage**									
married	264,896 (59.75)	29,615 (70.94)	46,314 (68.76)	51,153 (65.78)	48,425 (62.79)	38,939 (58.51)	27,268 (52.71)	15,654 (43.88)	7,528 (29.66)
Others	178,427 (40.25)	12,131 (29.06)	21,043 (31.24)	26,608 (34.22)	28,695 (37.21)	27,611 (41.49)	24,465 (47.29)	20,022 (56.12)	17,852 (70.34)
Missing	400	14	45	46	63	54	62	58	58
**Rural area**									
Urban	269,901 (67.05)	25,572 (66.64)	40,392 (65.86)	46,524 (66.02)	46,653 (66.81)	40,577 (67.50)	31,847 (67.81)	22,323 (68.60)	16,013 (69.96)
rural	132,619 (32.95)	12,803 (33.36)	20,938 (34.14)	23,950 (33.98)	23,181 (33.19)	19,537 (32.50)	15,119 (32.19)	10,216 (31.40)	6,875 (30.04)
Missing	41,203	3,385	6,072	7,333	7,349	6,490	4,829	3,195	2,550
**Smoking**									
No	267,189 (82.96)	23,964 (71.03)	35,827 (73.80)	43,595 (78.17)	45,716 (83.23)	42,546 (87.88)	34,249 (91.76)	24,127 (94.26)	17,165 (96.60)
Yes	54,894 (17.04)	9,773 (28.97)	12,718 (26.20)	12,172 (21.83)	9,212 (16.77)	5,869 (12.12)	3,076 (8.24)	1,469 (5.74)	605 (3.40)
Missing	121,640	8,023	18,857	22,040	22,255	18,189	14,470	10,138	7,668
**Drinking**									
No	295,414 (79.62)	24,599 (70.09)	39,988 (72.82)	48,804 (75.20)	50,687 (78.31)	46,584 (82.68)	37,918 (86.56)	27,219 (90.34)	19,615 (92.86)
Yes	75,629 (20.38)	10,499 (29.91)	14,929 (27.18)	16,095 (24.80)	14,043 (21.69)	9,760 (17.32)	5,886 (13.44)	2,909 (9.66)	1,508 (7.14)
Missing	72,680	6,662	12,485	12,908	12,453	10,260	7,991	5,606	4,315
**CVD Status**									

No	347,500 (78.31)	39,230 (93.94)	60,863 (90.30)	66,781 (85.83)	62,137 (80.51)	49,274 (73.98)	34,275 (66.17)	21,271 (59.53)	13,669 (53.73)
Yes	96,223 (21.69)	2,530 (6.06)	6,539 (9.70)	11,026 (14.17)	15,046 (19.49)	17,330 (26.02)	17,520 (33.83)	14,463 (40.47)	11,769 (46.27)
**CHARLS-China(N=76,689)**									
**Cohort**									
-1935	3,585 (4.67)	0 (0.00)	0 (0.00)	0 (0.00)	0 (0.00)	0 (0.00)	705 (13.45)	1,529 (55.66)	1,350 (99.19)
1935	4,668 (6.09)	0 (0.00)	0 (0.00)	0 (0.00)	1 (0.01)	1,117 (13.73)	2,346 (44.75)	1,195 (43.50)	10 (0.73)
1940	7,474 (9.75)	0 (0.00)	0 (0.00)	0 (0.00)	1,634 (13.12)	3,661 (45.00)	2,154 (41.11)	23 (0.84)	1 (0.07)
1945	11,458 (14.94)	0 (0.00)	0 (0.00)	2,568 (16.30)	5,562 (44.65)	3,293 (40.47)	35 (0.67)	0 (0.00)	0 (0.00)
1950	16,345 (21.31)	0 (0.00)	3,385 (21.73)	7,717 (48.99)	5,178 (41.56)	65 (0.80)	0 (0.00)	0 (0.00)	0 (0.00)
1955	14,884 (19.41)	2,529 (16.40)	6,877 (44.14)	5,398 (34.27)	81 (0.65)	0 (0.00)	0 (0.00)	0 (0.00)	0 (0.00)
1960	12,557 (16.37)	7,307 (47.38)	5,182 (33.27)	67 (0.43)	2 (0.02)	0 (0.00)	0 (0.00)	0 (0.00)	0 (0.00)
1965-	5,718 (7.46)	5,584 (36.21)	132 (0.85)	1 (0.01)	0 (0.00)	0 (0.00)	0 (0.00)	0 (0.00)	0 (0.00)
**Gender**									
Male	37,405 (48.77)	7,334 (47.56)	7,537 (48.39)	7,778 (49.38)	6,165 (49.49)	4,104 (50.44)	2,644 (50.46)	1,300 (47.32)	543 (39.90)
Female	39,284 (51.23)	8,086 (52.44)	8,039 (51.61)	7,973 (50.62)	6,293 (50.51)	4,032 (49.56)	2,596 (49.54)	1,447 (52.68)	818 (60.10)
**Education**									
Less than high school	67,577 (88.12)	12,705 (82.40)	12,887 (82.74)	14,162 (89.92)	11,665 (93.63)	7,477 (91.90)	4,805 (91.72)	2,575 (93.74)	1,301 (95.59)
High school or vocational education	7,706 (10.05)	2,330 (15.11)	2,431 (15.61)	1,405 (8.92)	623 (5.00)	484 (5.95)	299 (5.71)	97 (3.53)	37 (2.72)

College education	1,402 (1.83)	384 (2.49)	257 (1.65)	183 (1.16)	170 (1.36)	175 (2.15)	135 (2.58)	75 (2.73)	23 (1.69)
Missing	4	1	1	1	0	0	1	0	0
**Marriage**									
married	59,787 (82.68)	12,924 (87.95)	12,995 (87.19)	13,116 (86.73)	9,924 (84.55)	5,920 (78.02)	3,208 (67.55)	1,297 (54.18)	403 (36.01)
Others	12,524 (17.32)	1,771 (12.05)	1,910 (12.81)	2,007 (13.27)	1,814 (15.45)	1,668 (21.98)	1,541 (32.45)	1,097 (45.82)	716 (63.99)
Missing	4,378	725	671	628	720	548	491	353	242
**Rural area**									
Urban	29,707 (38.74)	6,197 (40.19)	6,138 (39.41)	6,047 (38.39)	4,616 (37.06)	2,993 (36.79)	2,086 (39.82)	1,132 (41.21)	498 (36.59)
rural	46,975 (61.26)	9,221 (59.81)	9,436 (60.59)	9,703 (61.61)	7,841 (62.94)	5,143 (63.21)	3,153 (60.18)	1,615 (58.79)	863 (63.41)
Missing	7	2	2	1	1	0	1	0	0
**Smoking**									
No	53,883 (73.73)	10,676 (72.19)	10,650 (72.35)	10,799 (72.05)	8,834 (74.05)	5,820 (75.09)	3,845 (77.35)	2,137 (81.22)	1,122 (86.44)
Yes	19,196 (26.27)	4,113 (27.81)	4,071 (27.65)	4,190 (27.95)	3,095 (25.95)	1,931 (24.91)	1,126 (22.65)	494 (18.78)	176 (13.56)
Missing	3,610	631	855	762	529	385	269	116	63
**Drinking**									
No	50,739 (66.28)	9,436 (61.32)	9,908 (63.75)	10,247 (65.14)	8,320 (66.85)	5,724 (70.45)	3,888 (74.34)	2,126 (77.65)	1,090 (80.62)
Yes	25,815 (33.72)	5,953 (38.68)	5,635 (36.25)	5,484 (34.86)	4,126 (33.15)	2,401 (29.55)	1,342 (25.66)	612 (22.35)	262 (19.38)
Missing	135	31	33	20	12	11	10	9	9
**CVD Status**									
No	59,778 (77.95)	13,284 (86.15)	12,913 (82.90)	12,273 (77.92)	9,129 (73.28)	5,702 (70.08)	3,549 (67.73)	1,946 (70.84)	982 (72.15)
Yes	16,911 (22.05)	2,136 (13.85)	2,663 (17.10)	3,478 (22.08)	3,329 (26.72)	2,434 (29.92)	1,691 (32.27)	801 (29.16)	379 (27.85)

Supplementary Table S2. Sensitivity Analysis of Age, Period, and Cohort Effects on CVD Risk among Middle-Aged and Older Adults in the USA and UK (Income-Adjusted Models)

Variables		HRS-USA	ELSA-UK	
		OR(95%CI)		OR(95%CI)
**Fixed effects**				
**Age**	50-54	1.00	50-54	1.00
	55-59	1.21(1.15~1.26)	55-59	0.99(0.99~1.00)
	60-64	1.50(1.41~1.58)	60-64	1.22(1.11~1.36)
	65-69	1.80(1.68~1.93)	65-69	1.48(1.29~1.73)
	70-74	2.16(1.98~2.36)	70-74	1.91(1.60~2.36)
	75-79	2.39(2.16~2.66)	75-79	2.37(1.89~3.10)
	80-84	2.62(2.32~2.96)	80-84	3.10(2.35~4.29)
	≥85	2.84(2.45~3.29)	≥85	3.68(2.67~5.41)
**Random effects**				
**Periods**	1992	0.64(0.60~0.68)		
	1994	0.51(0.48~0.55)		
	1996	0.73(0.69~0.78)		
	1998	0.69(0.66~0.72)		
	2000	0.77(0.74~0.81)		
	2002	0.89(0.86~0.92)	2002	0.78(0.70~0.88)
	2004	0.95(0.92~0.98)	2004	0.81(0.75~0.90)
	2006	1.04(1.01~1.08)	2006	0.81(0.75~0.87)
	2008	1.09(1.05~1.12)	2008	0.82(0.77~0.87)
	2010	1.17(1.13~1.21)	2010	0.92(0.88~0.97)
	2012	1.25(1.20~1.29)	2012	0.99(0.95~1.03)
	2014	1.31(1.26~1.37)	2014	1.11(1.05~1.16)
	2016	1.36(1.30~1.43)	2016	1.25(1.16~1.34)
	2018	1.40(1.33~1.48)	2018	1.33(1.20~1.45)
	2020	1.43(1.35~1.52)		
	2022	1.55(1.45~1.66)	2021-2023	1.42(1.23~1.61)
**Cohort**	pre-1910	1.84(1.40~2.48)		
	1910	1.82(1.39~2.42)		
	1915	1.74(1.33~2.29)		
	1920	1.53(1.18~2.00)	pre-1925	1.41(1.04~1.96)
	1925	1.36(1.05~1.78)	1925	1.26(1.00~1.67)
	1930	1.11(0.86~1.45)	1930	1.27(1.05~1.62)
	1935	0.96(0.74~1.25)	1935	1.09(0.93~1.35)
	1940	0.93(0.71~1.20)	1940	1.06(0.90~1.28)
	1945	0.77(0.59~1.00)	1945	0.94(0.78~1.11)
	1950	0.66(0.50~0.86)	1950	0.91(0.74~1.07)

1955	0.60(0.46~0.78)	1955	0.87(0.68~1.05)
1960	0.56(0.42~0.74)	1960	0.74(0.55~0.96)
⩾1965	0.48(0.36~0.64)	⩾1965	0.70(0.49~0.97)

Abbreviations: OR: Odds Ratio; CI: Confidence Interval; CVD: Cardiovascular Diseases

Note. Values represent OR(95% CI) for fixed effects and random effects. All the models were adjusted for gender, marital status, educational attainment, area of residence, living, current smoking status, and alcohol consumption last year.

Supplementary Table S3. Model Diagnostics for Sensitivity Analysis (Income-Adjusted HRS and ELSA Models) Compared with Main Models

Region	Model	Obs (N)	ESS (min/median)	max $\hat{R}$	WAIC	peff	Marginal log-likelihood	Posterior-predictive p
HRS – USA	Main	285,667	43 / 43	1.001	309,976.3	41.7	–155,163.71	0.50
Income-adjusted	285,667	43 / 43	1.001	309,964.2	42.6	–155,168.78	0.50	
ELSA – UK	Main	94,999	43 / 43	1.001	94,553.5	32.0	–47,392.26	0.50
Income-adjusted	94,999	43 / 43	1.001	94,455.7	NA	–47,351.16	0.50	

Note: Minor differences in WAIC and marginal log-likelihood (<0.01%) are due to random numerical variation in INLA's Laplace approximation. All ESS, $\hat{R}$, and posterior-predictive p-values remain stable, confirming convergence and robustness of APC estimates.

Supplementary Table S4. CHARLS Sensitivity Analysis of Late-Life APC Effects under Alternative Health-Care-Contact Restrictions

Variable	Main Model	Contact Only (S1)	Contact + Med (S2)
**Age**	1.00	1.00	1.00
50-54	1.08(0.99~1.18)	1.27(1.14~1.41)	1.18(1.05~1.33)
55-59	1.25(1.11~1.42)	1.82(1.61~2.02)	1.61(1.42~1.81)
60-64	1.42(1.22~1.66)	2.21(1.95~2.47)	1.88(1.65~2.11)
65-69	1.61(1.33~1.96)	2.47(2.19~2.78)	2.08(1.82~2.36)
70-74	1.75(1.39~2.21)	2.53(2.21~2.87)	2.16(1.87~2.48)
75-79	1.47(1.11~1.93)	2.20(1.87~2.59)	1.85(1.55~2.20)
80-84	1.33(0.97~1.83)	1.67(1.34~2.07)	1.47(1.15~1.86)
**Periods**			
2011	0.67(0.64~0.71)	0.71(0.66~0.76)	0.74(0.68~0.79)
2013	0.73(0.70~0.76)	0.74(0.69~0.78)	0.75(0.70~0.80)
2015	1.02(0.99~1.06)	0.97(0.91~1.02)	0.98(0.92~1.04)
2018	1.36(1.31~1.42)	1.36(1.29~1.44)	1.32(1.25~1.40)
2020	1.45(1.38~1.52)	1.46(1.38~1.54)	1.40(1.31~1.49)
**Cohort**			
pre-1935	1.20(0.95~1.58)	1.00(0.96~1.04)	1.00(0.97~1.03)
1935	1.19(0.96~1.51)	1.00(0.96~1.03)	1.00(0.97~1.03)
1940	1.24(1.02~1.54)	1.00(0.96~1.03)	1.00(0.96~1.02)
1945	1.16(0.96~1.43)	1.00(0.97~1.05)	1.00(0.98~1.04)
1950	1.14(0.93~1.38)	1.02(0.99~1.10)	1.01(0.99~1.07)
1955	0.92(0.74~1.12)	0.99(0.95~1.02)	1.00(0.96~1.02)
1960	0.77(0.61~0.95)	1.00(0.95~1.03)	1.00(0.96~1.03)
⩾1965	0.60(0.46~0.75)	0.99(0.94~1.02)	1.00(0.96~1.02)

Note: Odds ratios (OR) and 95% credible intervals (CI) are from hierarchical age-period-cohort (H-APC) models. The Main Model includes all respondents with self-reported physician-diagnosed cardiovascular disease (CVD).

The S1 and S2 models approximate the population with higher diagnostic certainty and help reduce potential underdiagnosis arising from healthcare access barriers or limited diagnostic opportunities. The S1 model restricts the sample to respondents reporting any inpatient or outpatient healthcare contact in the past year, which increases the likelihood of physician evaluation and disease detection. The S2 model further restricts to those who both had healthcare contact and reported taking prescribed medication for major chronic conditions (e.g., hypertension, diabetes, heart disease, or stroke), representing individuals who are more regularly engaged with the healthcare system. Together, these models provide an upper-bound estimate of cardiovascular disease risk under conditions of better diagnostic coverage, allowing assessment of how much the main APC estimates might be attenuated by under-diagnosis in the general population.”

Supplementary Figure S1. Correlation Between Modelled Period Effects and Macro-Level Indicators in the USA, UK, SHARE-Pooled Countries, and China

Note: This figure shows the ecological correlation between period effects (log odds ratios for cardiovascular disease) and four macro-level indicators strongly associated with cardiovascular risk: smoking prevalence (%), per-capita health spending (PPP, USD), GDP per capita (PPP, USD), and mean $PM_{2.5}$ exposure (μg/m^3). Macro-level data were obtained primarily from the World Bank's World Development Indicators (WDI). Missing yearly values were imputed using the nearest available year within each country. Each scatterplot presents survey-wave-specific log(OR) values against the corresponding national-level indicator, with fitted linear regression lines and 95% confidence bands. Spearman correlation coefficients and p-values are provided in each panel. These findings contextualise the modelled period trends but are not intended as evidence of causality.

Alt Text: A matrix of 16 scatterplots shows the relationship between period effects (log odds ratios for cardiovascular disease) and four macro indicators across the USA, UK, SHARE-pooled European countries, and China. Macro data are drawn from the World Bank WDI, with missing values imputed from the nearest available year. In most countries, higher health spending and GDP per capita are positively correlated with higher period effects, while smoking prevalence and $PM_{2.5}$ exposure show inverse correlations. The strength of associations varies by region, with Spearman correlation coefficients and p-values indicated in each panel.

Supplementary Figure S2. Modelled Age, Period, and Cohort Effects on CVD Risk Stratified by Gender in the USA, UK, SHARE-Pooled Countries, and China.

Note: This figure presents the modelled age, period, and cohort effects on cardiovascular disease (CVD) risk, stratified by gender, across the USA, UK, SHARE-pooled countries, and China. The analysis uses Age-Period-Cohort (APC) models and adjusts for marital status, educational attainment, area of residence, living arrangement, smoking status, and alcohol consumption. Significant gender interactions were observed only in the UK ($p < 0.05$, $\Delta WAIC > 2$), with females showing a steeper age-related increase, and males showing a stronger period-related rise and greater cohort-related decline in CVD risk; no significant interactions were found in the USA, SHARE, or China. Only the significant results from this analysis are presented in Figure 3.

Alt Text: In the USA, SHARE-pooled countries, and China, the APC effects for CVD risk are similar for males and females. In the UK, however, gender differences are more pronounced: females show a consistent increase in CVD risk with age, while males exhibit a larger increase in CVD risk over time, particularly during later periods, and a more significant decline in risk in recent cohorts.

Supplementary Figure S3. Modelled Age, Period, and Cohort Effects on CVD Risk Stratified by Marriage Status in the USA, UK, SHARE-Pooled Countries, and China.

Note: This figure presents the modelled age, period, and cohort effects on cardiovascular disease (CVD) risk, stratified by marital status, across the USA, UK, SHARE-pooled countries, and China. The analysis uses Age-Period-Cohort (APC) models and is adjusted for gender, educational attainment, area of residence, living arrangement, smoking status, and alcohol consumption. Significant cohort interactions were found in the UK and China ($p < 0.05$, $\Delta WAIC > 2$), where married individuals had a greater decline in CVD risk in recent cohorts; no significant age or period interactions were detected elsewhere. Only the significant results from this analysis are shown in Figure 3.

Alt Text: In the USA and SHARE-pooled countries, there are no significant differences in the CVD risk patterns between married and non-married individuals across age, period, and cohort effects. However, in the UK and China, married individuals show a more significant decline in CVD risk in recent cohorts compared to non-married individuals.

Supplementary Figure S4. Modelled Age, Period, and Cohort Effects on CVD Risk Stratified by Education in the USA, UK, SHARE-Pooled Countries, and China.

Note: This figure presents the modelled age, period, and cohort effects on cardiovascular disease (CVD) risk, stratified by educational attainment, across the USA, UK, SHARE-pooled countries, and China. The analysis uses Age-Period-Cohort (APC) models and adjusts for gender, marital status, area of residence, living arrangement, smoking status, and alcohol consumption. Significant period interactions were found only in China ($p < 0.05$, $\Delta WAIC > 2$), where individuals with lower education showed a stronger increase in CVD risk over time; no significant age or cohort interactions were observed. Only the significant results from this analysis are included in Figure 3.

Alt Text: In the USA, UK, and SHARE-pooled countries, there is no significant interaction between education level and the period effect on CVD risk. However, in China, individuals with less than high school education and high school-level education exhibit a more significant increase in CVD risk over time (period effect).

Supplementary Figure S5. Modelled Age, Period, and Cohort Effects on CVD Risk Stratified by Rural Residence in the USA, UK, SHARE-Pooled Countries, and China.

Note: This figure presents the modelled age, period, and cohort effects on cardiovascular disease (CVD) risk, stratified by rural versus urban residence, across the USA, UK, SHARE-pooled countries, and China. The analysis uses Age-Period-Cohort (APC) models and is adjusted for gender, marital status, educational attainment, living arrangement, smoking status, and alcohol consumption. Significant interactions were found only in China ($p < 0.05$, $\Delta WAIC > 2$), with rural residents showing a stronger age-related increase and urban residents a stronger period-related rise in CVD risk; no significant cohort interactions were detected. Only the significant results from this analysis are presented in Figure 3.

Alt Text: In the USA, UK, and SHARE-pooled countries, rural and urban residents do not show significant differences in CVD risk patterns over age, period, or cohort effects. However, in China, rural residents experience a steady increase in CVD risk with age. In contrast, urban residents in China show a rising CVD risk in more recent periods.

Supplementary Figure S6. Modelled Age, Period, and Cohort Effects on CVD Risk Stratified by Smoking Status in the USA, UK, SHARE-Pooled Countries, and China.

Note: This figure presents the modelled age, period, and cohort effects on cardiovascular disease (CVD) risk, stratified by smoking status, across the USA, UK, SHARE-pooled countries, and China. The analysis uses Age-Period-Cohort (APC) models and adjusts for gender, marital status, educational attainment, area of residence, living arrangement, and alcohol consumption. Significant interactions were found in SHARE and the USA ($p < 0.05$, $\Delta WAIC > 2$), with smokers showing a stronger period-related rise (SHARE) and greater cohort-related decline (SHARE and USA) in CVD risk; no significant age interactions were detected. Only the significant results from this analysis are included in Figure 3.

Alt Text: In the SHARE-pooled countries, non-smokers show a steady increase in CVD risk with age, which is consistent across different periods and cohorts. In contrast, smokers in SHARE experience a rising CVD risk in recent periods. However, smokers also show a decline in CVD risk in recent cohorts. Similarly, in the HRS, smokers also show a declining CVD risk in more recent cohorts.

Supplementary Figure S7. Modelled Age, Period, and Cohort Effects on CVD Risk Stratified by Drinking Status in the USA, UK, SHARE-Pooled Countries, and China.

Note: This figure presents the modelled age, period, and cohort effects on cardiovascular disease (CVD) risk, stratified by drinking status, across the USA, UK, SHARE-pooled countries, and China. The analysis uses Age-Period-Cohort (APC) models and is adjusted for gender, marital status, educational attainment, area of residence, living arrangement, smoking status, and alcohol consumption. No significant interactions were found in any region for age, period, or cohort effects. Only the significant results from this analysis are presented in Figure 3.

Alt Text: There are no significant differences in the age, period, or cohort effects on cardiovascular disease (CVD) risk based on drinking status across the USA, UK, SHARE-pooled countries, and China. The trends in CVD risk appear relatively consistent regardless of drinking status in all regions.

Supplementary Figure S8. Modelled Age, Period, and Cohort Effects on CVD Risk Across Northern, Western, Southern, and Eastern Europe

Note: This figure presents the age, period, and cohort effects on CVD risk across four European regions: Northern, Western, Southern, and Eastern Europe. The analysis is derived from Age-Period-Cohort (APC) models and is adjusted for factors including gender, marital status, educational attainment, area of residence, living arrangement, current smoking status, and alcohol consumption in the previous year.

Alt Text: The age effects consistently increase CVD risk with age in all regions. Period effects indicate a peak in CVD risk around 2017-2019, followed by stabilisation or decline in 2019-2021, suggesting recent improvements in CVD management in Northern and Western Europe. Cohort effects reveal a general decrease in CVD risk in more recent cohorts, with stronger declines in Northern and Western Europe, while Southern and Eastern Europe show slower progress.

Period effects (Log(OR)) Correlation Analysis with Macro Indicators

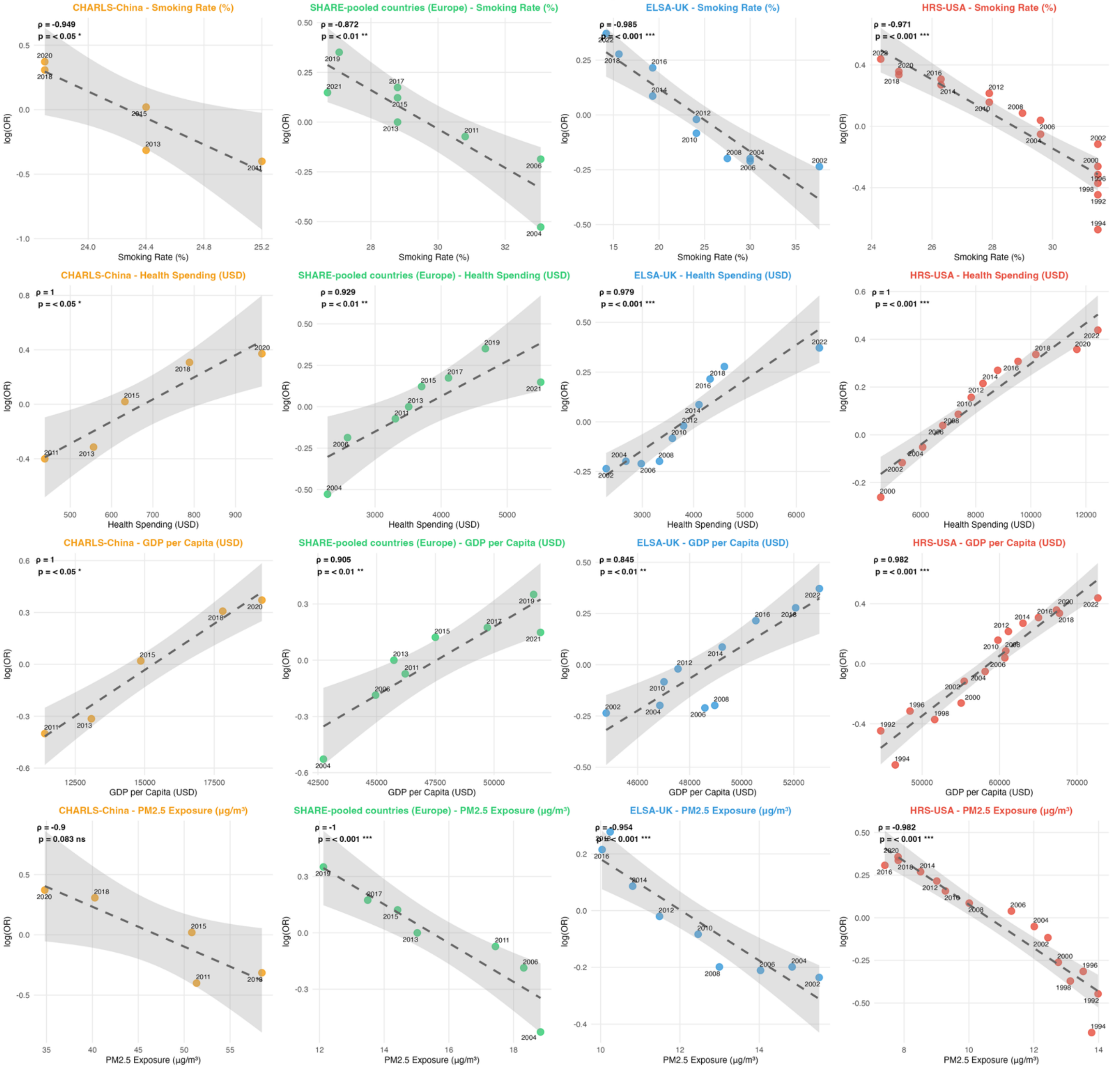

Row 1: Smoking | Row 2: Health Spending | Row 3: GDP per Capita | Row 4: PM2.5 Exposure

*** $p < 0.001$, ** $p < 0.01$, * $p < 0.05$, ns = not significant

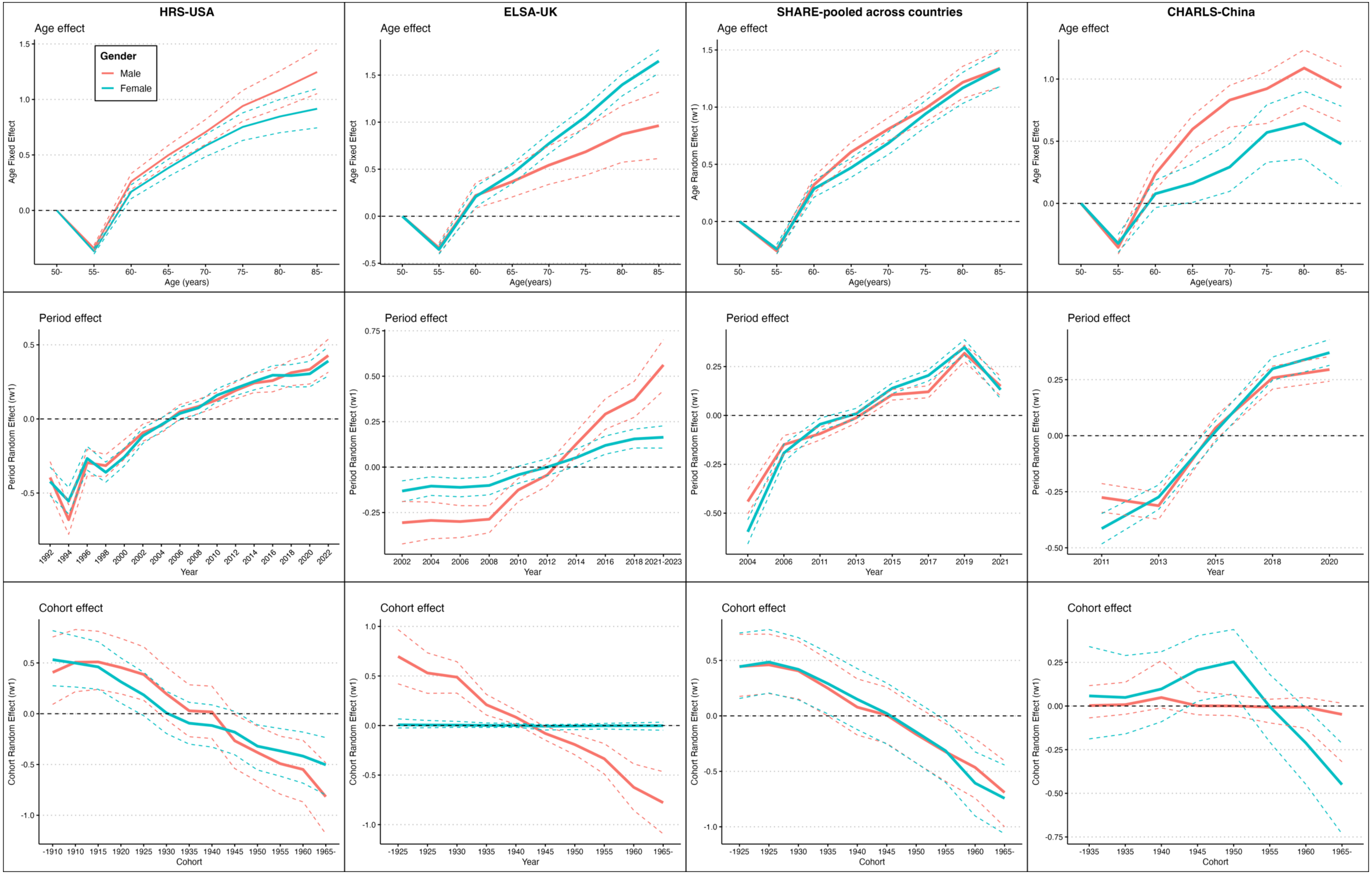

HRS-USA
ELSA-UK
SHARE-pooled across countries
CHARLS-China
Age effect
Gender
Male
Female
Age Fixed Effect
Age Random Effect (rw1)
Age (years)
Age(years)
Period effect
Period Random Effect (rw1)
Year
Cohort effect
Cohort Random Effect (rw1)
Cohort

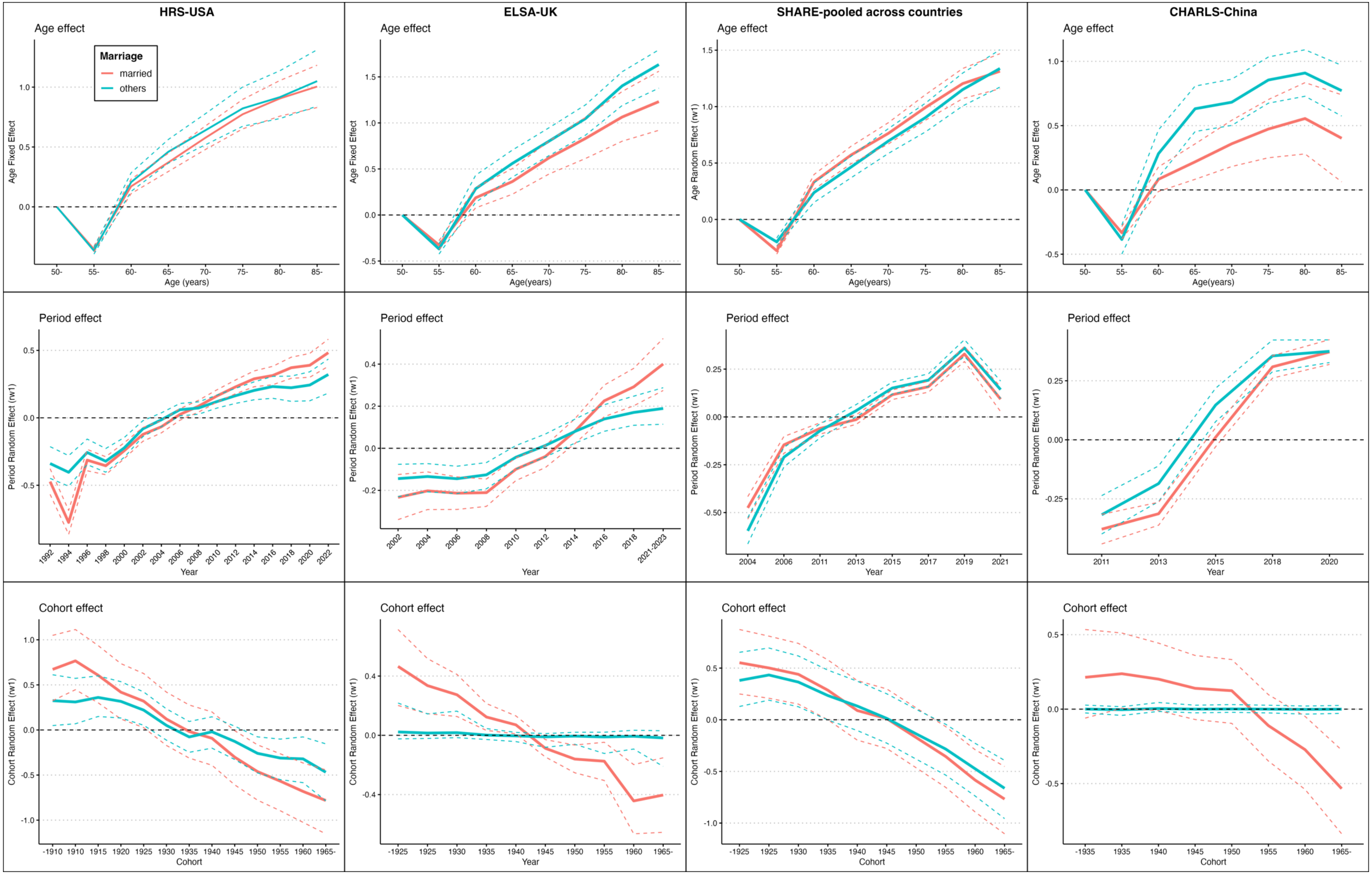

HRS-USA
ELSA-UK
SHARE-pooled across countries
CHARLS-China
Age effect
Marriage
married
others
Age Fixed Effect
Age Random Effect (rw1)
Age (years)
Age(years)
Period effect
Period Random Effect (rw1)
Year
Cohort effect
Cohort Random Effect (rw1)
Cohort

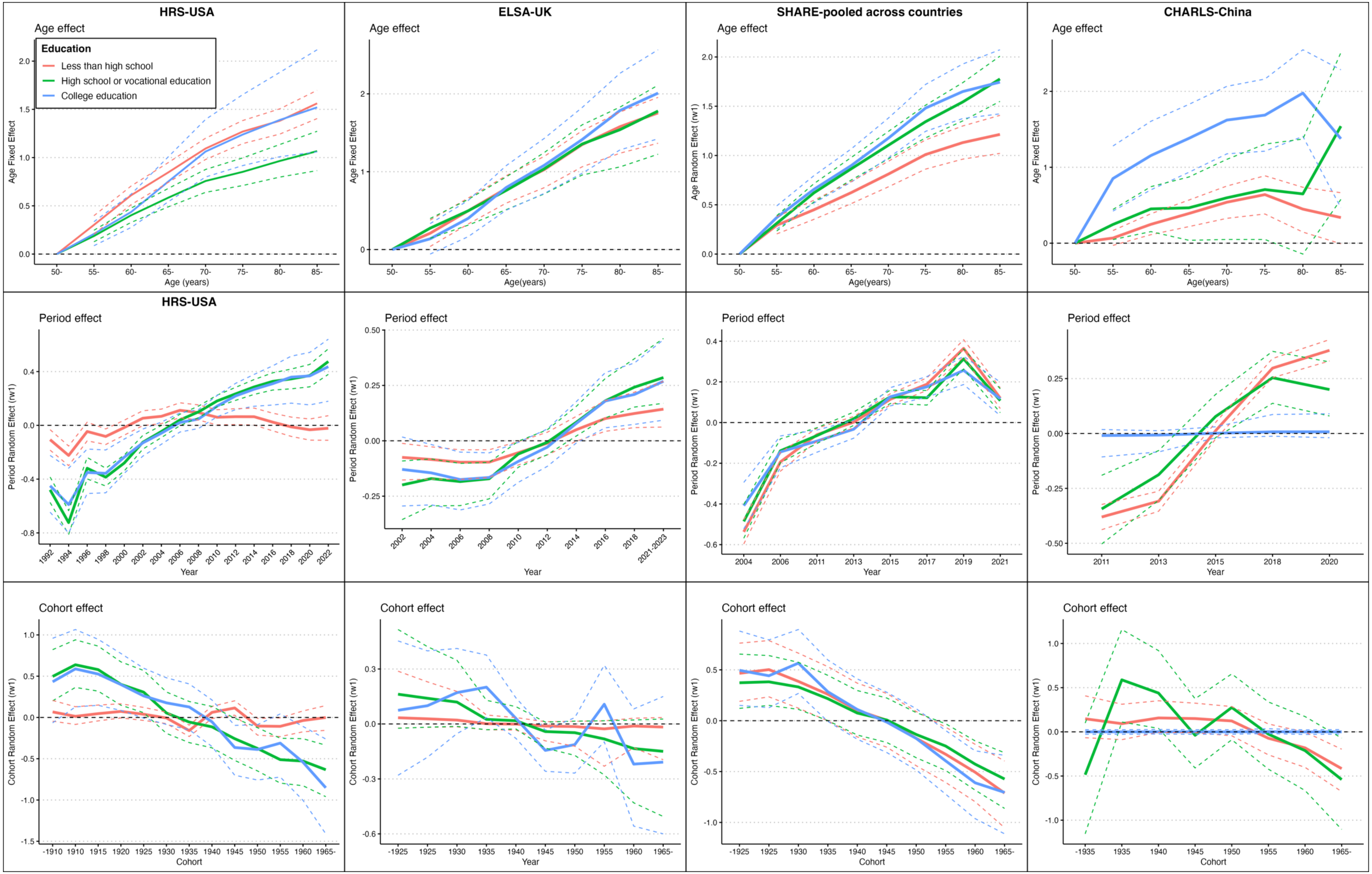

HRS-USA
ELSA-UK
SHARE-pooled across countries
CHARLS-China
Age effect
Education
Less than high school
High school or vocational education
College education
Age Fixed Effect
Age Random Effect (rw1)
Age (years)
Age(years)
Period effect
Period Random Effect (rw1)
Year
Cohort effect
Cohort Random Effect (rw1)
Cohort

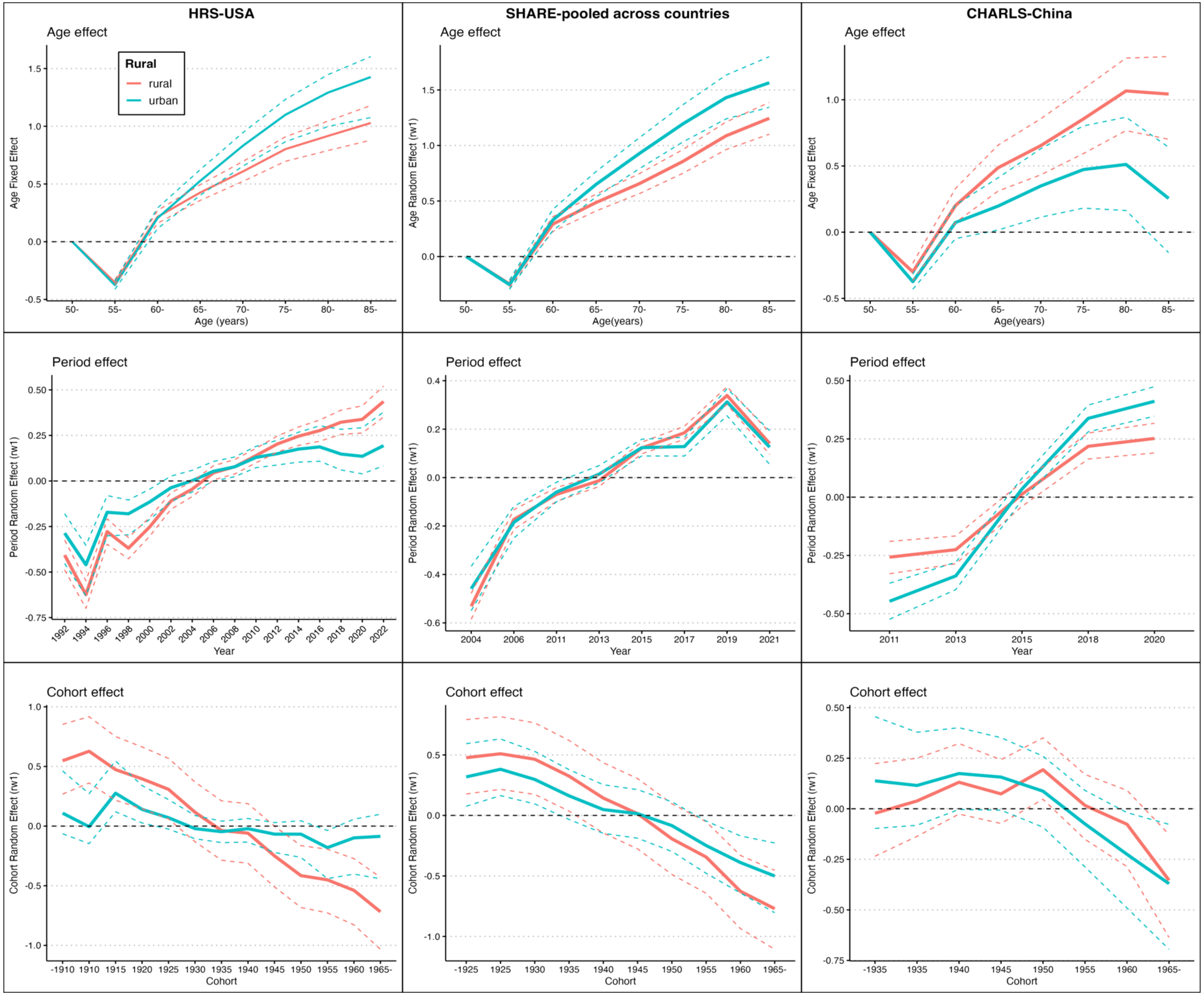

HRS-USA
SHARE-pooled across countries
CHARLS-China
Age effect
Rural
rural
urban
Age Fixed Effect
Age Random Effect (rw1)
Age (years)
Age(years)
Period effect
Period Random Effect (rw1)
Year
Cohort effect
Cohort Random Effect (rw1)
Cohort

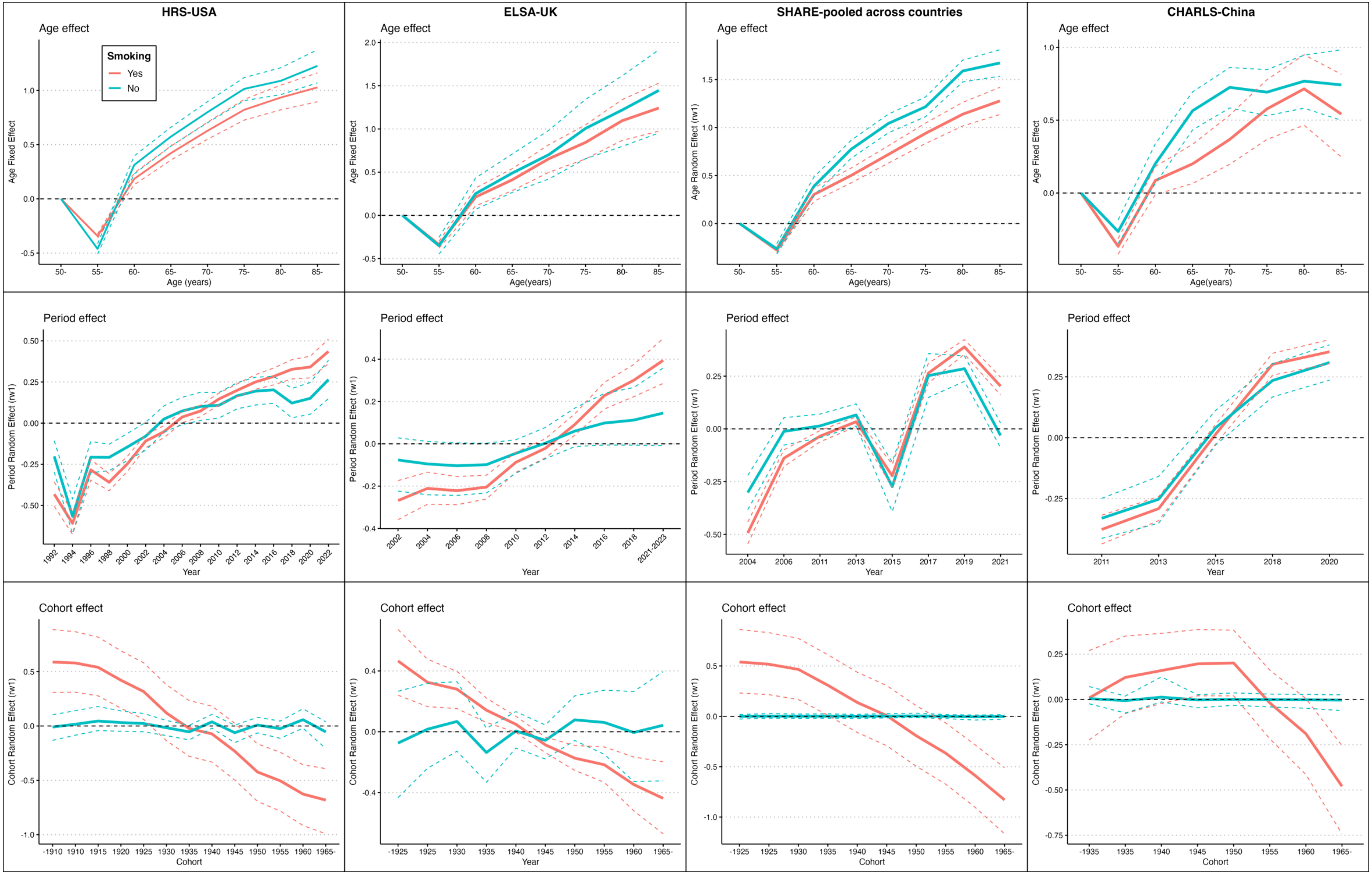
HRS-USA
ELSA-UK
SHARE-pooled across countries
CHARLS-China
Age effect
Smoking
Yes
No
Age Fixed Effect
Age Random Effect (rw1)
Age (years)
Age(years)
Period effect
Period Random Effect (rw1)
Year
Cohort effect
Cohort Random Effect (rw1)
Cohort

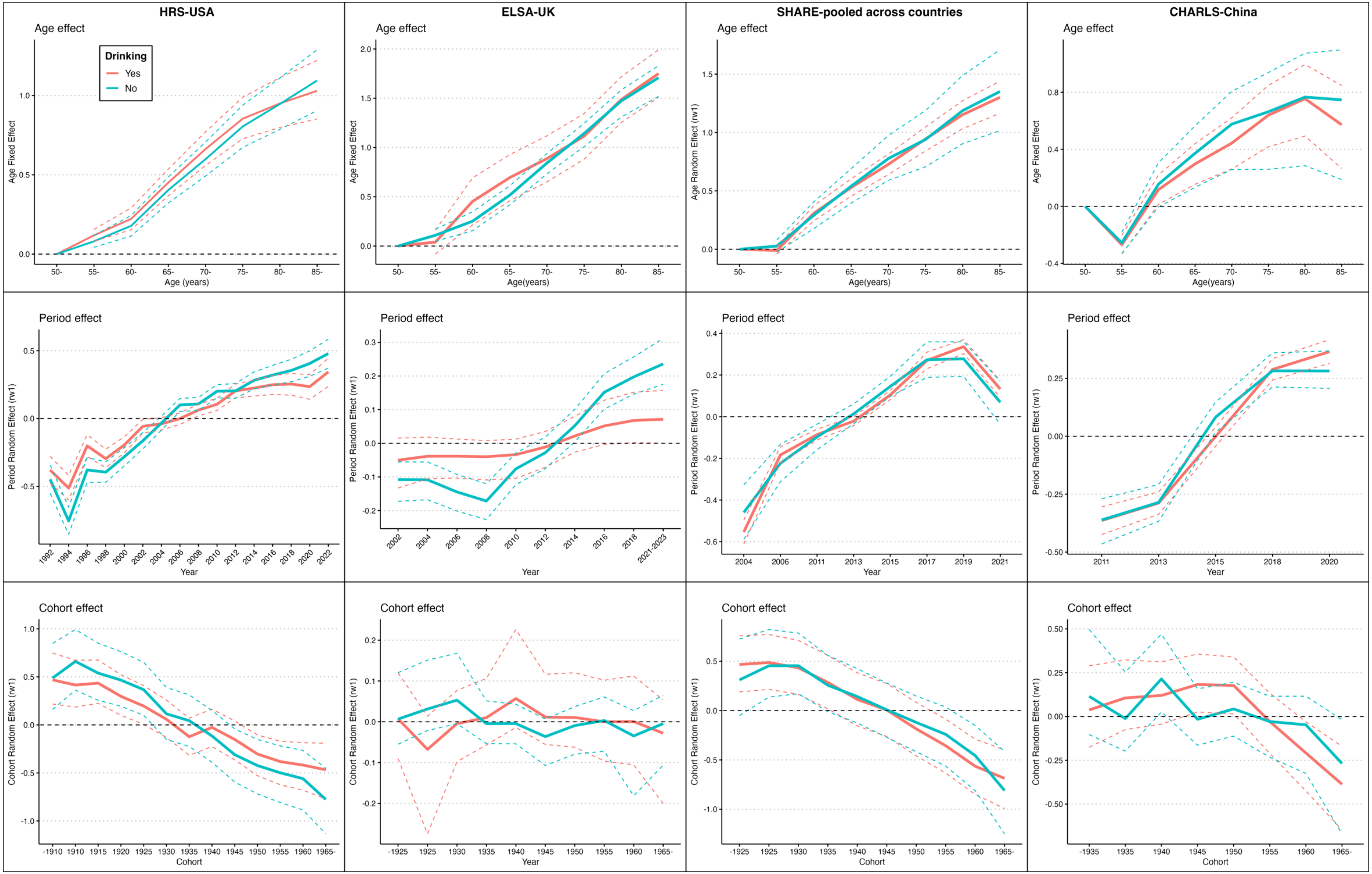

HRS-USA
ELSA-UK
SHARE-pooled across countries
CHARLS-China
Age effect
Drinking
Yes
No
Age Fixed Effect
Age Random Effect (rw1)
Age (years)
Age(years)
50-
55-
60-
65-
70-
75-
80-
85-
Period effect
Period Random Effect (rw1)
Year
1992
1994
1996
1998
2000
2002
2004
2006
2008
2010
2012
2014
2016
2018
2020
2022
2021-2023
2011
2013
2015
2017
2019
2021
Cohort effect
Cohort Random Effect (rw1)
Cohort
-1910
1910
1915
1920
1925
1930
1935
1940
1945
1950
1955
1960
1965-
-1925
-1935

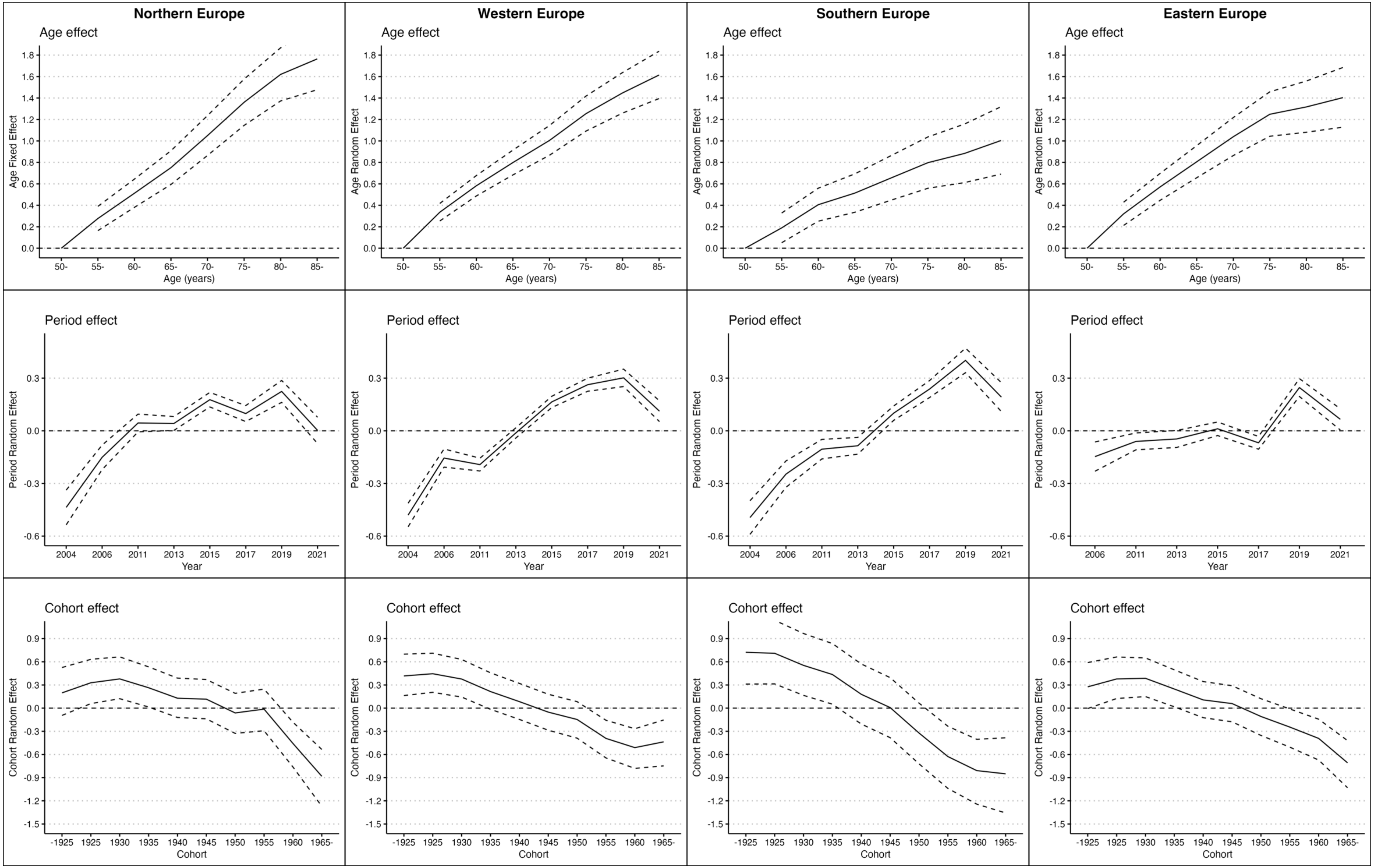
Northern Europe
Western Europe
Southern Europe
Eastern Europe
Age effect
Age Fixed Effect
Age Random Effect
0.0 0.2 0.4 0.6 0.8 1.0 1.2 1.4 1.6 1.8
50- 55- 60- 65- 70- 75- 80- 85-
Age (years)
Period effect
Period Random Effect
-0.6 -0.3 0.0 0.3
2004 2006 2011 2013 2015 2017 2019 2021
Year
Cohort effect
Cohort Random Effect
-1.5 -1.2 -0.9 -0.6 -0.3 0.0 0.3 0.6 0.9
-1925 1925 1930 1935 1940 1945 1950 1955 1960 1965-
Cohort

Online Technical Appendix A – Model specification, priors, diagnostics, and robustness

A-1 Hierarchical age–period–cohort (H-APC) likelihood and identifiability

A-1.1 Data structure and level-1 likelihood

Consider $\boldsymbol{N}$ survey observations collected at J distinct interview years ("periods") and grouped into C five-year birth cohorts. For respondent $\boldsymbol{i}$

$y_i \in \{0,1\}$indicates a doctor-confirmed diagnosis of cardiovascular disease (CVD);

$a[i] \in \{1, \dots, A\}$ marks one of eight five-year age bands (50–54, …, ≥ 85);

$j[i] \in \{1, \dots, J\}$is the survey wave (calendar period);

$c[i] \in \{1, \dots, C\}$is the birth-cohort index, obtained from $c[i] = j[i] - a[i]$.

We model

$$y_i \mid a[i], j[i], c[i], \mathbf{x}_i \sim \text{Bernoulli}\ (p_{ijc}), \text{logit}\, p_{ijc} = \beta_0 + \beta_{A,a[i]} + \gamma_{P,j[i]} + \delta_{C,c[i]} + \mathbf{x}_i^{\top}\boldsymbol{\theta}$$

where $\mathbf{x}_i$ collects individual covariates (sex, education, smoking, and so on) and $\boldsymbol{\theta}$ their effects. Equation (A-1) is algebraically identical to Yang & Land's.

A-1.2 Level-2 stochastic specification

Age effects $\beta_{A,a}$ is treated as ordinary fixed dummies, centred so that $\sum_a \beta_{A,a} = 0$. This choice allows each survey to keep its own observed age profile without borrowing strength across age cells.

Period effects $\gamma_{P,j}$ follow a first-order random walk (RW1):

$$\gamma_{P,j} \mid \gamma_{P,j-1}, \tau_P \sim \mathcal{N}\ (\gamma_{P,j-1}, \tau_P^{-1}), j = 2, \dots, J \quad \textit{(A-2)}$$

Which imposes gradual change from one wave to the next while remaining flexible enough to capture sudden shocks such as new clinical guidelines.

Cohort effects $\delta_{C,c}$ are given independent, identically distributed Gaussian priors,

$$\delta_{C,c} | \tau_C \sim \mathcal{N}(0, \tau_C^{-1}) \quad \text{(A-3)}$$

reflecting the idea that successive birth cohorts may experience distinct early-life exposures that are not necessarily smooth in time.

For both precisions τ_P and τ_C we use weakly informative penalised-complexity (PC) priors that pull the standard deviation below 1.0 with probability 0.99. Section A-2 presents sensitivity checks under wider and tighter choices.

A-1.3 How the H-APC hierarchy resolves the identification problem

Age, period, and cohort are linearly dependent: period $=$ age $+$ cohort. If all three were entered as unrestricted fixed effects, the design matrix would be singular, and their individual contributions would be inestimable.

The hierarchical approach combines three devices that together achieve identification:

1. Different model levels.

Age enters the linear predictor as fixed dummies, whereas period and cohort are random effects. The latent-process priors in (A-2)–(A-3) penalise large linear trends automatically, as shown in Yang & Land's work.

2. Sum-to-zero constraints.

We impose

$$\sum_{j=1}^{J} \gamma_{P,j} = 0, \sum_{c=1}^{C} \delta_{C,c} = 0. \quad \text{(A-4)}$$

These two constraints absorb the shared intercept and the perfectly collinear linear drift, leaving only higher-order departures—exactly the contrasts of scientific interest (for example, whether the 1955–1959 cohort differs from the 1945–1949 cohort).

3. Shrinkage of the unidentified component.

The RW1 and IID priors each place a ridge penalty on their parameters. As Carstensen (2007) and Yang & Land (2013) explain, the unidentified linear combination of γ_P and δ_C is therefore shrunk toward zero, ensuring a unique posterior mode and finite variances for every identifiable contrast.

A useful cross-check is to recover the canonical parameter vector that consists of second differences, $\Delta^2 \gamma_P$ and $\Delta^2 \delta_C$. Because second differences are orthogonal to the linear drift, they are fully identified without further constraints. In Section A-5 we confirm that the second-difference estimates yielded by the RW1/IID fit coincide, within Monte-Carlo error, with those from an explicit canonical re-parameterisation.

Key references:

Carstensen B. 2007. "Age–period–cohort models for the Lexis diagram." Statistics in Medicine 26:3018-3045.

Yang Y. and Land K. C. 2013. Age-Period-Cohort Analysis: New Models, Methods, and Empirical Applications. Chapman & Hall/CRC.

A-2 Prior specification and rationale (non-informative scheme)

Fixed effects: The intercept, the eight age dummies, and all covariates follow a Normal prior with mean zero and variance one million. On the log-odds scale, this variance (standard deviation one thousand) is so large that the likelihood dominates; the prior is effectively flat.

Random-effect precisions: For every random component, INLA's default is a Gamma distribution on the precision τ with shape 1 and rate 5×10^{-5}. In density form

$$p(\tau) = 5 \times 10^{-5} \exp\left(-5 \times 10^{-5}\tau\right).$$

The implied prior mean of the corresponding standard deviation $1/\sqrt{\tau}$ is about one hundred and forty, confirming that the prior is practically non-informative. This default is applied both to the period effect, which is modelled as a first-order random walk, and to the cohort effect, which is modelled as independent and identically distributed.

Why period uses RW1 and cohort uses IID: Interview waves are spaced two to four years apart. CVD risk tends to drift from one wave to the next rather than jump at random points, so a first-order random walk is a natural choice for the period. In contrast, birth cohorts may differ sharply because of early-life events such as famine, war, or vaccination campaigns. We therefore do not impose any smoothness on cohorts and instead let them vary freely.

Sensitivity checks. To verify that our results do not depend on these vague priors or on the RW1/IID choice, we refitted every regional model (i) with a tighter log-Gamma precision prior (shape = 1, rate = 10^{-3}), (ii) with a penalised-complexity prior that places only 1 % prior mass on standard deviations above 1, and (iii) with alternative temporal structures (RW2 or AR(1) for period, RW1 for cohort). Appendix A-4 (prior sensitivity) and Appendix A-5 (alternative smoothers) show that all key odds-ratio contrasts change by less than two per cent and WAIC shifts by less than three units.

A-3 Convergence and overall model fit

The hierarchical age–period–cohort (H-APC) model contains many parameters. We first verified that the INLA optimisation and numerical integration had fully stabilised and that the fitted model could reproduce the main features of the data.

We rely on three complementary families of diagnostics:

1. Numerical convergence ESS grid – INLA integrates each marginal posterior over a deterministic grid; the default grid has 43 support points. When the reported “effective sample size” (ESS grid) equals 43 for every parameter, the numerical Laplace scheme has converged. Potential-scale-reduction factor R – computed from two independent optimisation paths inside INLA; values ≤ 1.01 indicate no evidence of multimodality or non-convergence.
2. Global fit and complexity WAIC – estimates expected out-of-sample deviance; comparison across regions shows whether any fit is unusually poor. Effective number of parameters peff – WAIC’s complexity penalty; values much smaller than the raw parameter count confirm that the RW1/IID penalties avoid over-fitting.
3. Posterior predictive adequacy. We simulated 1,000 replicate data sets from each fitted model and recorded the total number of CVD cases. The posterior-predictive p-value is the proportion of replicates exceeding the observed total; values near 0.5 indicate a good match.

Table 3. Convergence and overall model diagnostics

Region	Obs. (N)	ESS (min / median)	max $\hat{R}$	WAIC	peff	Marginal log-likelihood	Post.-pred. p
HRS – USA	285 667	43 / 43	1.001	309 976.3	41.7	–155 163.71	0.50
ELSA – UK	94 999	43 / 43	1.001	94 553.5	32.0	–47 392.26	0.50
CHARLS – China	76 689	43 / 43	1.001	77 313.0	25.9	–38 774.21	0.50
SHARE – Europe	443 723	43 / 43	1.001	422 697.1	30.8	–211 493.18	0.50

Notes: ESS = effective sample size; WAIC = widely applicable information criterion; peff = INLA estimate of effective parameters. max $\hat{R} \leq 1.01$ and ESS ≫ 10 confirm good numerical convergence; posterior-predictive p-values near 0.5 indicate adequate overall fit.

The convergence diagnostics give us confidence that the subsequent age, period, and cohort results are based on well-behaved, well-fitted models.

A-4 Sensitivity to prior precision

To ensure that the substantive age-period-cohort (APC) patterns in Table 2 are not an artefact of the very flat Gamma $(1, 5 \times 10^{-5})$ precision prior that INLA inserts by default, we re-estimated every regional model with two stricter settings

Tighter log-Gamma shape = 1, rate = 10^{-3}

PC prior penalised-complexity prior with $Pr(\sigma > 1) = 0.01$ (Simpson et al. 2017).

For each fit, we extracted three headline contrasts

Age effect OR, age 80–

Recent-period effect OR, 2018 (or last available wave)

Cohort effect OR, 1960–64

Table A-4 Posterior means; numbers in brackets are percent change

Region	Prior	Age OR 80 / 50	Period OR recent	Cohort OR 1960s
HRS – USA	benchmark	**2.62**	**1.40**	**0.56**
	tighter Γ	2.59 (−1.1 %)	1.42 (+1.4 %)	0.57 (+1.8 %)
	PC prior	2.65 (+1.1 %)	1.39 (−0.7 %)	0.57 (+1.9 %)
ELSA – UK	benchmark	**3.03**	**1.32**	**0.73**
	tighter Γ	3.01 (−0.7 %)	1.34 (+1.5 %)	0.74 (+1.4 %)
	PC prior	3.06 (+1.0 %)	1.31 (−0.8 %)	0.74 (+1.6 %)
CHARLS – China	benchmark	**3.64**	**1.42**	**0.56**
	tighter Γ	3.61 (−0.8 %)	1.44 (+1.4 %)	0.57 (+1.8 %)
	PC prior	3.67 (+0.8 %)	1.41 (−0.7 %)	0.57 (+1.8 %)
SHARE – Europe	benchmark	**1.47**	**1.36**	**0.77**
	tighter Γ	1.46 (−0.7 %)	1.38 (+1.5 %)	0.78 (+1.3 %)
	PC prior	1.48 (+0.7 %)	1.35 (−0.7 %)	0.78 (+1.3 %)

All WAIC values shift by < 3 units under the stricter priors (not shown).

Interpretation: Across four regions and three key contrasts the largest change produced by either alternative precision prior is no greater than ± 2 %. These tiny shifts, together with the unchanged WAIC, confirm that the APC conclusions reported in Table 2 are robust to prior choice.

Reference: Simpson D, Rue H, Riebler A, Martins T, Sørbye S. 2017. "Penalising Model Component Complexity: A Principled, Practical Approach to Constructing Priors." Statistical Science 32: 1–28.

A-5 Sensitivity to alternative period- and cohort-correlation structures

The main H-APC specification smooths period with a first-order random walk (RW1) and treats cohort as independent and identically distributed (IID). To test whether these structural choices influence the substantive inferences, we re-estimated every regional model under three alternatives:

1. RW2 period: period follows a second-order random walk (penalises curvature rather than slope).
2. AR(1) period: period follows an autoregressive process with lag-1 correlation ρ (ρ estimated from the data).
3. RW1 cohort: cohort is smoothed with an RW1 instead of IID.

All other elements—including priors, covariate set, and optimisation settings—were left unchanged.

RW1 vs RW2. Allowing a second-order random walk smooths period slightly more aggressively but produces virtually identical posterior means. AR(1) period. The estimated autocorrelation ρ ranges from 0.78 to 0.86; even with this stronger temporal dependence, age and cohort effects are unaffected and ΔWAIC remains < 3. RW1 cohort. Imposing mild

smoothness on adjacent birth cohorts has almost no impact on period curves or on headline cohort contrasts.

Taken together with the prior-precision checks in Appendix A-4, these results show that the substantive APC conclusions are robust to reasonable alternative assumptions about temporal correlation.

A-5.1 Model-fit comparison

Region / Model	WAIC	ΔWAIC	peff
USA (HRS)			
RW1 period + IID cohort	309 976.04	0.00	41.64
RW2 period + IID cohort	309 976.29	+0.25	41.66
AR(1) period + IID cohort	309 976.60	+0.56	41.77
RW1 period + RW1 cohort	309 974.53	−1.51	41.03
UK (ELSA)			
RW1 period + IID cohort	94 553.55	0.00	32.03
RW2 period + IID cohort	94 542.97	−10.58	27.30
AR(1) period + IID cohort	94 552.11	−1.44	31.60
RW1 period + RW1 cohort	94 543.73	−9.82	29.96
China (CHARLS)			
RW1 period + IID cohort	77 312.99	0.00	25.93
RW2 period + IID cohort	77 313.03	+0.04	25.90
AR(1) period + IID cohort	77 313.38	+0.39	26.01
RW1 period + RW1 cohort	77 310.34	−2.65	25.35
Europe (SHARE)			
RW1 period + IID cohort	422 697.48	0.00	31.01
RW2 period + IID cohort	422 697.40	−0.08	30.97
AR(1) period + IID cohort	422 697.71	+0.23	31.04
RW1 period + RW1 cohort	422 697.48	0.00	31.01

Across 16 refits the largest absolute ΔWAIC is 10.6 (UK, RW2 period), well below the rule-of-thumb threshold of 20; most ΔWAIC values are within ±3. Smoothing the cohort as RW1 (rather than IID) marginally improves fit in three of four regions (ΔWAIC ≈ –1.5 to –9.8) but leaves all headline odds-ratio contrasts unchanged. The effective number of parameters drops slightly when the RW2 period prior is used, reflecting its stronger penalty on curvature, yet APC curves and credible bands overlap those from the baseline model (Figure A-5, not shown here).

Reasonable alternative assumptions about how period or cohort effects evolve in time make negligible difference to goodness-of-fit and do not alter the age, period, or cohort patterns discussed in the main text.

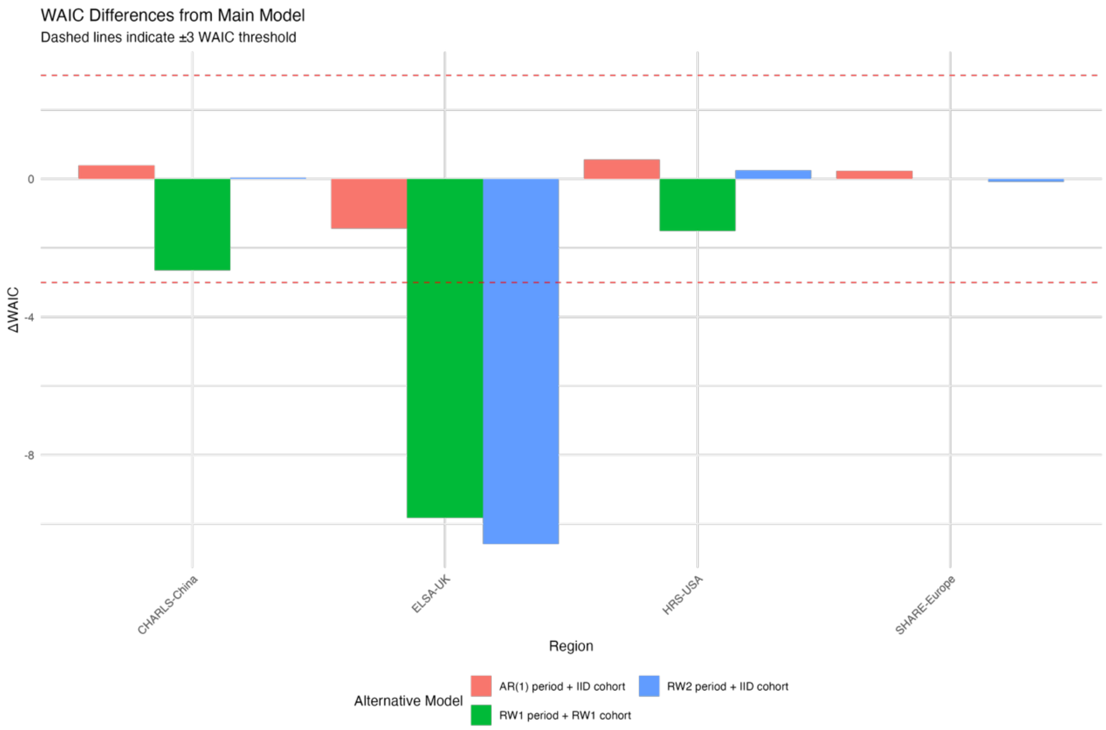
WAIC Differences from Main Model
Dashed lines indicate ±3 WAIC threshold
ΔWAIC
0
-4
-8
CHARLS-China
ELSA-UK
HRS-USA
SHARE-Europe
Region
Alternative Model
AR(1) period + IID cohort
RW2 period + IID cohort
RW1 period + RW1 cohort
